# Fractal analysis of brain shape formation predicts age and genetic similarity in human newborns

**DOI:** 10.1038/s41593-025-02107-w

**Published:** 2025-12-29

**Authors:** Stephan Krohn, Amy Romanello, Nina von Schwanenflug, Jerod M. Rasmussen, Claudia Buss, Sofie L. Valk, Christopher R. Madan, Carsten Finke

**Affiliations:** 1 Humboldt-Universität zu Berlin, Institut für Philosophie, 10099, Berlin, Deutschland; 2 Charité — Universitätsmedizin Berlin, Corporate Member of Freie Universität Berlin, Humboldt-Universität zu Berlin, and Berlin Institute of Health (BIH), Department of Neurology with Experimental Neurology, Berlin, Germany; 3 Development, Health and Disease Research Program, University of California, Irvine, Irvine, CA USA; 4 Department of Pediatrics, University of California, Irvine, Irvine, CA USA; 5 Charité − Universitätsmedizin Berlin, Corporate Member of Freie Universität Berlin, Humboldt-Universität zu Berlin, and Berlin Institute of Health (BIH), Institute of Medical Psychology, Berlin, Germany; 6 German Center for Child and Adolescent Health (DZKJ), Partner Site Berlin, Charité − Universitätsmedizin Berlin, Berlin, Germany; 7 German Center for Mental Health (DZPG), Partner Site Berlin, Charité − Universitätsmedizin Berlin, Berlin, Germany; 8 Lise Meitner Research Group NeuroBioSocial, Max Planck Institute for Human Cognitive and Brain Sciences, Leipzig, Germany; 9 Institute of Neuroscience and Medicine (INM-7: Brain and Behavior), Research Centre Jülich, Jülich, Germany; 10 Institute of Systems Neuroscience, Heinrich Heine University Düsseldorf, Düsseldorf, Germany; 11 School of Psychology, University of Nottingham, Nottingham, UK

**Keywords:** Development of the nervous system, Computational neuroscience

## Abstract

The neonatal period represents a critical phase of human brain development. During this time, the brain shows a dramatic increase in size, but how its morphology emerges in early life remains largely unknown. Here we show that human newborns undergo a rapid formation of brain shape, beyond the expected growth in brain size. Using fractal dimensionality (FD) analysis of structural neuroimaging data, we show that brain shape strongly reflects infant maturity beyond differences in brain size, significantly outperforms brain size in predicting infant age at scan (mean error approximately 4 days), detects signatures of premature birth that are not captured by brain size, is systematically more sensitive to genetic variability among infants and is superior in predicting which newborns are twin siblings, with up to 97% accuracy. Additionally, FD captures age and genetic information significantly better than earlier morphological measures, including cortical thickness, curvature, gyrification, sulcation, surface area and the T1-weighted/T2-weighted ratio. These findings identify the formation of brain shape as a fundamental maturational process in human brain development and show that, biologically, FD should be interpreted as a developmental marker of early-life brain maturity, which is rooted in geometry rather than size.

## Abstract (web summary)

Fractal analysis of structural MRI captures the formation of brain shape in human newborns and outperforms other morphological measures in predicting infant age and genetic similarity, including the identification of twins from their brain data.

## Main

The human brain undergoes profound morphological changes over the lifespan^1–3^, developing from a small and smooth structure in utero to the complex, highly convoluted structure that characterizes mature brains. Non-invasive studies with structural magnetic resonance imaging (MRI) have facilitated great progress in understanding these age-related morphological changes, aided by the increasing availability of large open-access datasets of human MRI recordings^4,5^.

These developments have recently led to the first normative trajectories of human brain structure over the lifespan, similar to growth charts of body weight or height^1^. In a complementary approach, a recent framework uses structural neuroimaging data to predict brain age from modeled trajectories of healthy brain aging, revealing clinically meaningful discrepancies between apparent brain age and true chronological age in a variety of developmental and adult disorders^6^.

Although these advances have yielded important insights into structural brain changes from childhood to senescence, large-scale investigations of perinatal brain development have remained limited, not least owing to the technical and ethical challenges of acquiring MRI data from human fetuses and newborns^1,3,7^. Such investigations are vital, however, as perinatal brain maturation is fundamental for the development of cognitive capacities, and, in turn, this period represents a critical window of vulnerability for later cognitive deficits and neurodevelopmental disorders^3,8–10^.

To overcome this gap, recent collaborative efforts such as the developing Human Connectome Project (dHCP) now provide the opportunity to study perinatal brain development in curated datasets of unprecedented size, quality and accessibility^11^. These resources are met by parallel advances in the processing of early-life neuroimaging data, including neonatal brain atlases^12–14^ and the adaptation of well-established processing pipelines to the specificities of the newborn brain regarding size variability and tissue contrasts^14,15^.

Concurrently, powerful new methodologies have emerged that capture the shape characteristics of the human brain from structural MRI, moving beyond information reflected by measures of brain size such as volume.

To illustrate why shape-related measures can capture additional features of brain morphology, consider the example of a fictitious structure of 10,000 voxels. By definition, the volume estimate of this structure is given by the voxels it consists of (and yields 10 ml if voxels are 1-mm^3^ isotropic). Clearly, however, there are many ways in which these voxels could be arranged in space, resulting in different morphological constellations or ‘shapes’ of the structure. In this regard, a recent line of research has shown that such shape characteristics are reliably captured by a structure’s fractal dimensionality (FD)^16–18^. In brief, FD stems from a branch of mathematics that investigates the spatial scaling properties of geometric objects, showing that the traditional notions of Euclidean dimensions (that is, 1 for a line, 2 for a plane and 3 for a cube) do not apply well to objects of the biophysical world^19^. Instead, natural objects often show a high degree of involvedness, yielding irregular shapes that exhibit non-Euclidean scaling properties (see Methods). Such irregular scaling is more adequately described by a non-integer fractal dimension (from Latin ‘fractus’: broken, fragmented or irregular), which expresses scaling properties that lie in between the idealized dimensions of Euclid and can be viewed as a measure of the object’s structural complexity^16,19–22^.

In neuroscience, fractal analysis of structural MRI has provided researchers with a new tool to study brain shape empirically, yielding FD as a highly age-sensitive neuroimaging phenotype^16,17,21–23^. On the technical side, previous studies showed that FD is robustly calculated from MRI segmentations of various modalities^16,17^, shows better test−retest reliability than volumetric measures of brain morphology^18^ and is applicable to all tissue compartments of the brain—including cortical gray matter (GM), white matter (WM) and subcortical regions^16,17,23,24^—as FD can be estimated from any voxel-indexed segmentation mask. The latter also distinguishes FD from other shape-related measures such as gyrification, whose application is typically limited to the cortical sheet. Notably, FD has been shown to outperform both thickness and gyrification in capturing the age-related variance of cortex morphology in later life^17^, suggesting that FD maps unique morphological signatures beyond these earlier measures. Moreover, FD has not only proven sensitive to age-related brain changes in healthy individuals^17,23,25–27^ but also detects morphological alterations in a variety of clinical conditions, including neurodevelopmental disorders^28,29^.

In the present work, we leverage these advances to study how the shape of the human brain develops in very early life. Specifically, we apply fractal analysis to the neonatal dHCP data and assess (1) the cross-sectional, longitudinal and predictive capacity of brain shape to reflect infant age; (2) the impact of key developmental factors on brain shape, including sex, singleton versus multi-fetal pregnancy and premature birth; and (3) the relationship between brain shape variability and genetic variability across individual newborns. Therein, we compare FD against both volume as a measure of brain size and common surface-derived measures of brain morphology, showing that fractal analysis systematically outperforms these earlier measures in capturing infant age, the morphological variability of individual brains and genetic information.

## Results

### Quantifying brain shape in human newborns

**Fig. 1:**
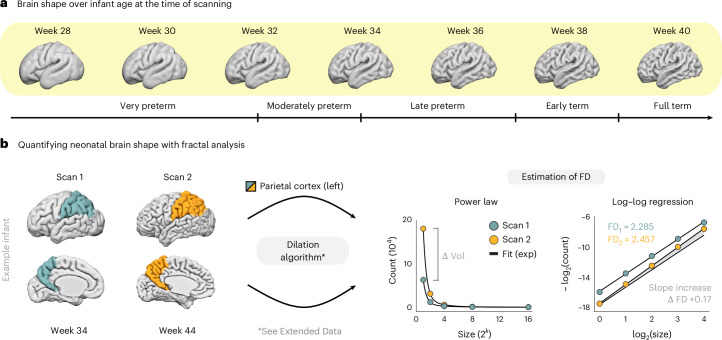
Quantifying brain shape in human newborns. **a**, Differences in brain shape over infant age at the time of scanning, illustrated for cortical GM of the left hemisphere. Surface renderings correspond to the age-specific group averages of the dHCP data. Maturity levels follow the criteria by the WHO^30^ and the ACOG^31^. **b**, Quantifying neonatal brain shape with FD. The FD estimate is calculated from a dilation procedure of the voxel-indexed segmentation mask^17,18^, which measures the scaling properties of the structure through iterative convolution with varying spatial kernels (see Extended Data Fig. 1 for an illustration). Scaling behavior is assessed by the power law relationship between kernel size and the count of scaled measurement units after convolution. The slope of this relationship in log−log space then yields the structure’s FD estimate. This estimation is illustrated for the left parietal cortex of an exemplary infant born at 32.6 weeks and scanned at 34 weeks and 44 weeks post-menstrual age. Over this 10-week interval, the morphological change of the region (left) is reflected by an increase in the structural complexity estimate (right). exp, exponential; Vol, volume.

Here we analyze structural MRI scans from the third dHCP release^11^, which includes 782 human neonates and covers a wide range of infant maturity levels (27−45 weeks post-menstrual age). Figure 1a visualizes the differences in cortical morphology over these varying degrees of maturity, as defined by the age criteria of the World Health Organization (WHO)^30^ and the American College of Obstetricians and Gynecologists (ACOG)^31^.

**Extended Data Fig. 1:**
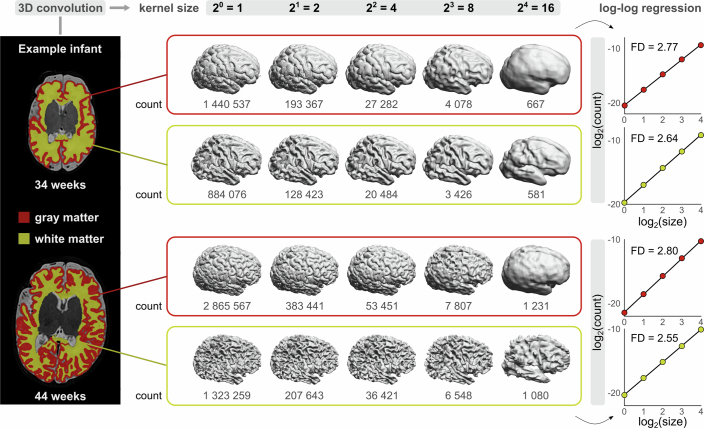
Illustration of the dilation algorithm to estimate fractal dimensionality (FD) from the scaling properties of a voxel-indexed segmentation mask. The left panel displays representative voxel-indexed slices of global tissue segmentations of T2-weighted data in the dHCP. These data correspond to the same exemplary infant of main Fig. 1b, scanned once at 34 and once at 44 weeks of age. The classes of cortical gray matter (red) and white matter (yellow) are contrasted for visualization. The middle panels display surface renderings of the respective 3D masks after convolution with spatial kernels of increasing size (‘dilation’), which represent the physical scales over which the FD estimate is computed (see Methods). The latter correspond to a range of voxel sizes expressed as 2^*k*^, with *k* = 0, 1, …, 4, following previous applications^17,18,23,26^. For each of these spatial scales, a 3D convolution of the tissue mask is calculated, resulting in a volumetric count in relation to the spatial scale. The FD estimate is then given by the slope of the relationship between these counts and the kernel sizes in log-log-space, which is illustrated for the example data in the right column. The FD changes from scan 1 to scan 2 thus correspond to changes in the scaling properties of the respective structures (independent of their absolute size).

To quantify these shape differences, we apply fractal analysis with a dilation algorithm that estimates the spatial scaling properties of a brain structure from its voxel-indexed three-dimensional segmentation mask (Methods and Extended Data Fig. 1). In brief, through iterative convolution of this mask with a set of spatial kernels, one estimates the power law relationship between the size of the scales and the count of scaled measurement units, where the FD estimate is given by the slope of this relationship in log−log space^16–18^. Figure 1b illustrates this procedure for the left parietal cortex of an exemplary infant scanned shortly after birth at 34 weeks of age and once again at 44 weeks. Over this 10-week interval, the morphological change that is visible from the surface renderings (left) is reflected by an increase in structural complexity from baseline to follow-up (right).

### Brain shape reflects infant maturity beyond differences in brain size

**Fig. 2:**
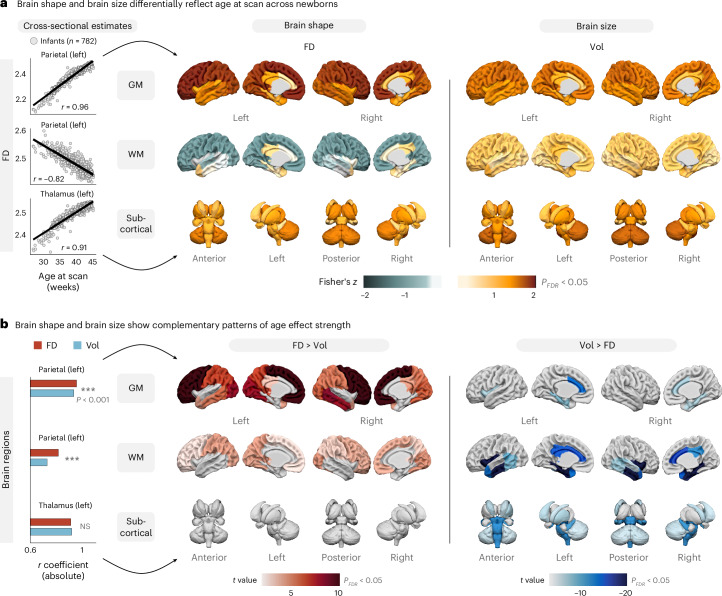
Brain shape reflects infant maturity beyond differences in brain size. **a**, Cross-sectional correlations between infant age at scan and FD as a measure of brain shape (left) and volume (Vol) as a measure of brain size (right; two-tailed product−moment correlation tests). Correlation coefficients were Fisher *z*-transformed and thresholded to *P* < 0.05 after false discovery rate (FDR) adjustment. **b**, Region-wise comparison of age effects (two-tailed Williams’ test of absolute effect size; example regions: parietal GM left, *P*_FDR_ = 2.3 × 10^−10^; parietal WM left, *P*_FDR_ = 2.7 × 10^−5^; thalamus left: *P*_FDR_ = 0.10). For color-coded regions, the null hypothesis that FD and Vol are equally strongly correlated with age was rejected at *P* < 0.05 after FDR adjustment. Higher age correlations for brain shape are shown on the left; higher age correlations for brain size are shown on the right. Note that, in some regions (for example, the thalamus), infant age was reflected equally strongly by both measures. NS, not significant. Source data

**Extended Data Fig. 2:**
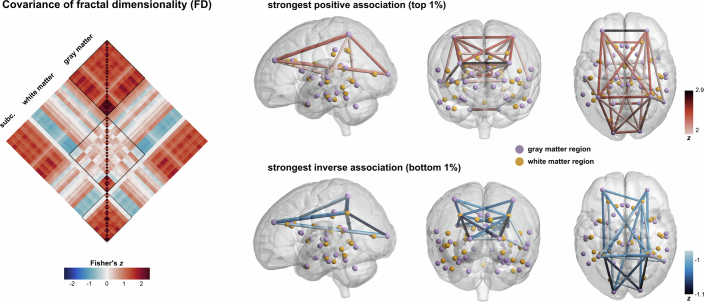
Covariance network of fractal dimensionality (FD). The network displays the region-to-region covariance of fractal dimensionality values across individual newborns (n = 782, as in main Fig. 2a). The left-hand side displays the direction of how brain regions covary with each other, showing primarily positive associations within regions of the same tissue compartment as well as for cortical gray matter and subcortical areas, while several white matter regions show inverse associations to subcortical areas and cortical gray matter. The right-hand side shows this network in brain space, thresholded to the strongest 1% of positive and inverse associations, respectively. Here, the strongest positive associations (top) are observed between areas of the same tissue class and homologous areas, and the strongest inverse associations (bottom) between cortical gray matter and white matter areas.

First, we related cross-sectional differences in infant age to the structural complexity and the size of each brain region, measured by FD and volume, respectively. Therein, older infants showed significantly higher FD across cortical GM and subcortical areas, paralleled by inverse age effects across several WM areas (Fig. 2a, left). This GM − WM difference was corroborated by the covariance across infants (Extended Data Fig. 2), where FD values covaried in the same direction for homologous regions across hemispheres but were inversely related in several GM and WM regions.

**Extended Data Fig. 3:**
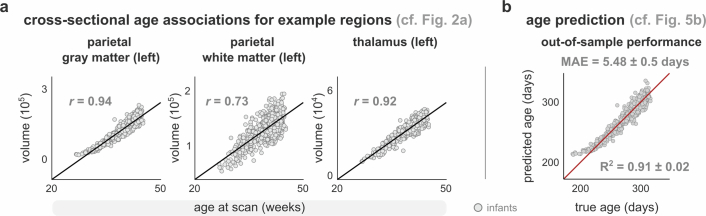
Illustration of age analyses for volume. **a**, Illustration of cross-sectional age-volume associations for the same three example regions of main text Fig. 2a. **b**, Out-of-sample performance of age prediction from volumes, evaluated using a 10-fold cross-validation scheme in analogy to main text Fig. 5b. The red line represents the identity line (that is, perfect prediction). Note the shift towards overestimating age in very premature infants, which was not observed in age prediction from fractal dimensionality. MAE: mean absolute prediction error in days; R^2:^ variance explained in the test data.

Conversely, age−volume associations were strictly positive (Fig. 2a, right), such that brain structures were universally larger in older neonates, as is expected from a continuous postnatal growth in brain size (see Extended Data Fig. 3a for analogous plots of the example regions). Although effect sizes were generally large for both measures, directly comparing age−FD and age−volume effects revealed a complementary spatial pattern, in which FD tracked infant age more strongly across most cortical GM and WM areas (Fig. 2b, left), whereas volume showed larger effect sizes in temporal, cingulate and some subcortical areas (Fig. 2b, right).

**Extended Data Fig. 4:**
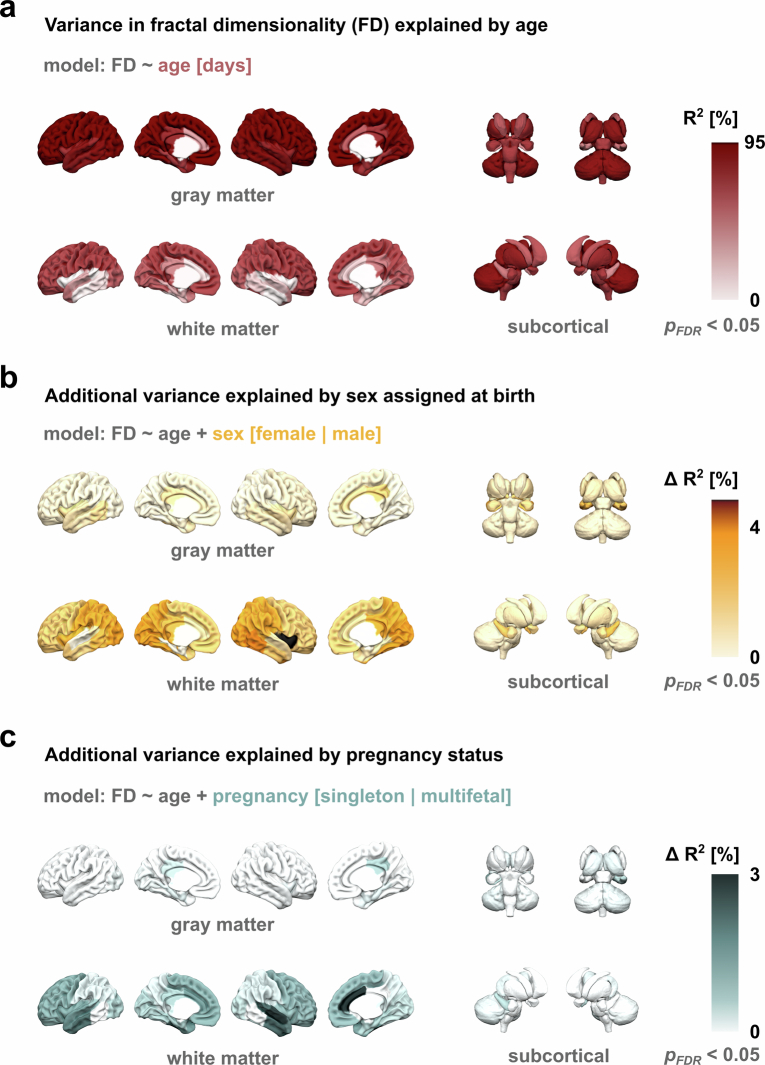
Explaining variance in brain shape with age, sex and pregnancy status. **a**, Variance in fractal dimensionality (FD) explained by age at scan in a compact linear regression model. **b**, Hierarchical regression results showing the additional variance explained (ΔR^2^) by including the sex of the infant in the model. **c**, Hierarchical regression results showing the additional variance explained by including pregnancy status (singleton or multifetal). *P*-values are derived from *F*-tests for linear models and FDR-adjusted over regions.

Furthermore, we investigated how neonatal brain shape is influenced by infant sex and pregnancy status (singleton versus multi-fetal). Region-wise hierarchical regression confirmed strong age−FD effects across the entire brain (Extended Data Fig. 4a) but also revealed an additional impact of sex and pregnancy status on FD, albeit on a smaller scale (up to 5% additional variance explained). These effects were most pronounced in WM areas and showed spatial clusters, with infant sex primarily influencing parietal, occipital and insular WM, as well as the hippocampus, and pregnancy effects clustering in frontal, temporal and cingulate WM (Extended Data Fig. 4b,c).

### FD outperforms surface-derived measures in capturing the age-related variability of the neonatal cortex

**Fig. 3:**
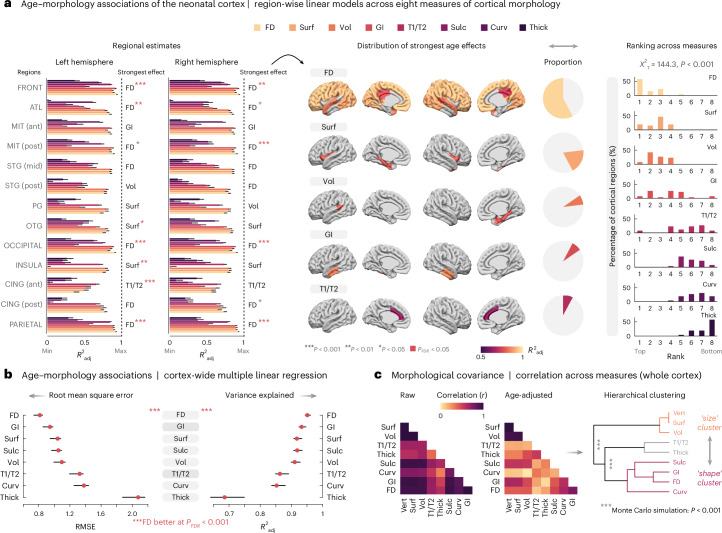
FD outperforms earlier morphological measures in capturing the age-related variability of the neonatal cortex. **a**, Age−morphology associations of the neonatal cortex across eight measures: cortical thickness (Thick), curvature (Curv), gyrification index (GI), sulcation (Sulc), surface area (Surf), T1w/T2w ratio (T1/T2), volume (Vol) and FD. The left panels show the adjusted *R*^2^ values (*R*^2^_adj_) from linear regression models against age at scan for each measure and cortical region (*n* = 609 infants; 26 regions based on the modified ALBERT atlas^12^; error bars: upper bound of bootstrapped 95% confidence intervals). For each region, the model with the highest *R*^2^_adj_ was statistically compared to the second-best model with a permutation test. Abbreviations indicate the measure with the highest-ranking *R*^2^_adj_ in each region, with asterisks reflecting the outcome of the permutation tests (***P < 0.001; **P < 0.01; *P < 0.05; red: *P*_FDR_ < 0.05 over all regions). The middle panel maps the spatial distribution of strongest (raw) effects across the entire cortex; the right panel visualizes the relative ranking of age effects across all regions and measures, compared with a Kruskal−Wallis test (*P* = 6.3 × 10^−28^). **b**, Whole-cortex models of age−morphology associations. The plots show the root mean square error (RMSE) in weeks (left) and the *R*^2^_adj_ (right) obtained from multiple linear regression models including all cortical regions for the same *n* = 609 infants as above. Permutation tests showed lower RMSE and higher *R*^2^_adj_ for FD compared to all others measures at *P*_FDR_ < 0.001. Error bars correspond to bootstrapped 95% confidence intervals. **c**, Morphological covariance across cortical features. Here, measures were estimated for the whole (unparcelled) cortex and included the number of vertices from surface modeling (Vert) as a further control measure, as the latter is expected to correlate perfectly with Surf. The matrices show raw (left) and age-adjusted (middle) correlations across measures. The latter was subjected to hierarchical clustering (right), where the three main clusters were assessed with a Monte Carlo simulation (sigclust test; first branch: *P* = 3.0 × 10^−13^, second branch: *P* = 4.2 × 10^−9^). For exact *P* values not listed here, see the Supplementary Information. Region labels are as follows: ATL, anterior temporal lobe; CING, cingulate; FRONT, frontal; MIT, medial-inferior temporal gyrus; OTG; occipitotemporal gyrus; PG, parahippocampal gyrus; STG, superior temporal gyrus. Source data

As both FD and volume are derived from a voxel-wise three-dimensional representation of the brain, the above findings raise the additional question of how FD compares to morphological measures that are derived from surface modeling^14,15,32^. Therefore, we additionally compared FD against cortical thickness, curvature, gyrification, sulcation, surface area and the T1-weighted/T2-weighted (T1w/T2w) ratio and asked how closely each of these measures captures the age-related variability of the neonatal cortex. Specifically, we first employed region-wise linear models to estimate which measure yields the highest adjusted coefficient of determination (*R*^2^_adj_) in each cortical area (Fig. 3a) and compared the highest-ranking model to the respective second-best model with a permutation approach. Therein, FD showed the strongest age associations in more cortical regions than all other measures combined (highest-ranking: 15/26 regions (58%); univariate permutation test: 11/14 regions (79%); false discovery rate (FDR) adjusted: 8/11 regions (73%)). Overall, FD thus showed the strongest and anatomically most comprehensive associations with age (Fig. 3a, middle), making it the top-ranking measure, followed by surface area and volume (Fig. 3a, right). Similarly, when all cortical regions were considered together in a multiple linear regression approach, FD showed the lowest root mean square error and highest variance explained across all measures (*P*_FDR _< 0.001), followed by gyrification and surface area (Fig. 3b). Moreover, we studied how FD relates qualitatively to these other measures by estimating their ‘morphological covariance’ across the whole cortex. Overall, measures were strongly correlated with each other across infants of different ages (Fig. 3c, left), which is intuitive as all these features are expected to develop largely in parallel. However, age adjustment of these data revealed a suggestive qualitative pattern (Fig. 3c, middle), in which measures are sorted into a ‘size’ cluster (including volume) on the one hand and a ‘shape’ cluster (including FD) on the other hand (Fig. 3c, right). Notably, FD’s closest neighbor in this tree was gyrification, arguably the most shape-sensitive among the alternative measures of cortex morphology.

### Longitudinal development of brain shape in individual newborns

**Fig. 4:**
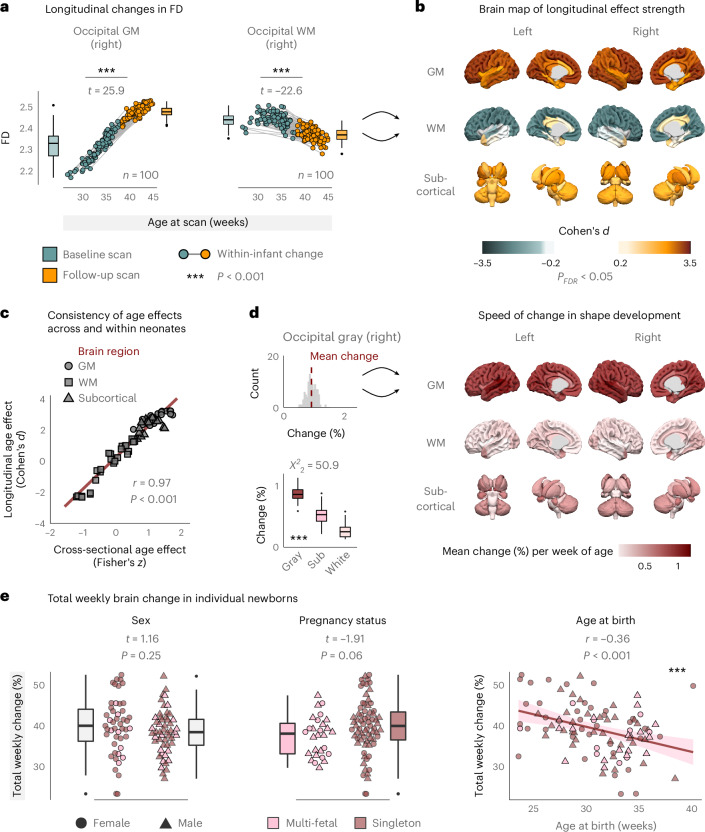
Longitudinal development of brain shape in individual newborns. **a**, Longitudinal changes in FD within individual infants, illustrated for occipital GM (*P* = 7.3 × 10^−46^) and WM (*P* = 7.8 × 10^−41^) of the right hemisphere. Repeated scans were available for *n* = 100 newborns. *t*-statistics were derived from paired *t*-tests (two-sided) between baseline and follow-up scans. **b**, Whole-brain distribution of longitudinal age effects. Cohen’s *d* was derived from the region-wise *t*-tests (two-sided), FDR adjustment over regions. **c**, Product−moment correlation between cross-sectional age effects (Fisher’s *z*; Fig. 2) and longitudinal effect sizes over individual brain regions (two-sided, *P* = 7.7 × 10^−41^). **d**, Quantifying the speed of shape developments. The upper-left panel illustrates the change per additional week of age for right occipital GM, where the histogram reflects individual infants. The brain map displays the mean weekly change derived from these distributions for all brain regions. The lower-left image shows the distributions of weekly change over tissue classes. *χ*^2^ statistic from Kruskal−Wallis test (*P* = 8.7 × 10^−12^). Pairwise comparisons between tissue classes with Dunn’s test are significant at *P*_FDR_ ≤ 0.002. **e**, Total weekly change of brain shape in individual newborns (*n* = 100 as above), compared by sex, pregnancy status and age at birth (*P* = 2.2 × 10^−4^; error band: 95% confidence interval for predictions from a linear model). Boxes in **a**, **d** and **e** display the interquartile range (IQR; lower hinge: 25th percentile; upper hinge: 75th percentile; center line: median), and whiskers cover the furthest data points within 1.5× IQR. Source data

Next, we investigated how brain shape develops within individual newborns. To this end, we analyzed the longitudinal FD trajectories in all infants for whom repeated scans were available (*n* = 100). Figure 4a illustrates these trajectories for occipital GM and WM of the right hemisphere. Therein, all infants showed a pronounced increase in FD for occipital GM (paired *t*-test: *t*_99_ = 25.9, *P* < 0.001), paralleled by a simultaneous decrease in the corresponding WM region (*t*_99_ = −22.6, *P* < 0.001; Fig. 4a), with large effect sizes for both (occipital GM: Cohen’s *d* = 3.2; WM: Cohen’s *d* = −2.2). Mapping these longitudinal developments across the whole brain revealed systematic FD increases in cortical GM and subcortical areas, with simultaneous decreases across several WM areas (Fig. 4b).

The spatial pattern of longitudinal age effects thus strongly resembled the distribution of cross-sectional age effects (Fig. 2a). Indeed, explicitly comparing these estimates showed that the spatial pattern of age−FD associations was virtually identical across and within individual newborns (*r* = 0.97, *P* < 0.001; Fig. 4c).

To characterize the spatial specificity of these dynamics, we furthermore estimated the speed of development as the relative change that a brain region exhibits per additional week of age. The upper-left inset of Fig. 4d illustrates this rate of change for the right occipital GM of individual infants, and the average speed per region is plotted in the brain map (Fig. 4d, right). Notably, the speed of shape development showed significant differences across tissue classes (Kruskal−Wallis: *χ*^2^_2_ = 50.9, *P* < 0.001; Fig. 4d, lower-left inset), with cortical GM developing fastest, followed by an intermediate speed in subcortical areas and WM areas showing the slowest change with age (all pairwise comparisons *P*_FDR_ ≤ 0.002).

Furthermore, we analyzed the total weekly brain change within each newborn to study how developmental factors influence individual longitudinal trajectories (Fig. 4e). Therein, we observed no difference in the speed of development between female and male neonates (*t* = 1.16, *P* = 0.25) nor between singleton and multi-fetal pregnancies (*t* = −1.91, *P* = 0.06). Interestingly, however, total weekly brain change was negatively associated with age at birth (*r* = −0.36, *P* < 0.001), such that the brains of more prematurely born infants showed a higher rate of change compared to infants who were born later (Fig. 4e, right).

### Explaining the tissue-specific direction of age−FD effects

The above analyses thus revealed a consistent spatial pattern of age−FD effects, which was observed both cross-sectionally and longitudinally and in which more mature brains are characterized by higher GM−FD and lower WM−FD. To explain this tissue-specific effect direction, we conducted six follow-up analyses. Here we summarize the main results, but see the Supplementary Information for details.

**Extended Data Fig. 5:**
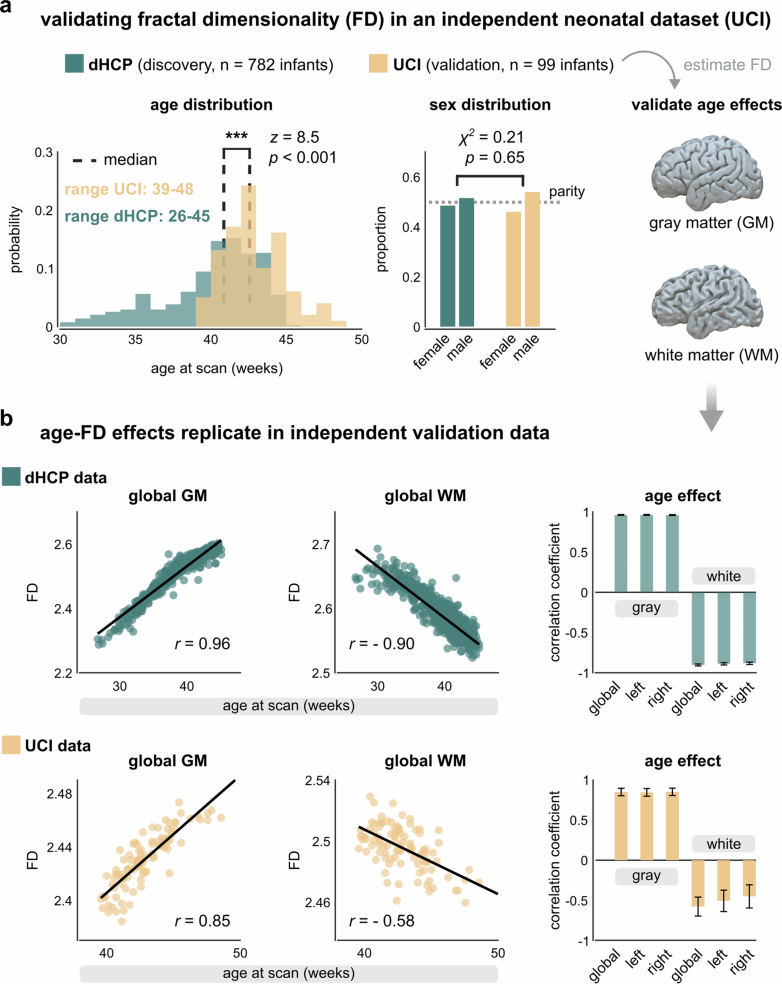
The directionality of age-FD effects replicates in external validation data. **a**, Illustration of the datasets used for replication analyses. The dHCP data corresponds to the discovery dataset reported throughout the main manuscript, including 782 unique newborns. The validation dataset corresponds to a neonatal cohort from the University of California, Irvine (UCI), including 99 newborn infants. The sex ratio was not different between the two cohorts (*χ*^2^ test, *P* = 0.645), whereas UCI infants were significantly older (rank-sum test, *P* = 2.5*10^−17^). To harmonize comparisons, we analyzed global segmentations of cortical gray matter (GM) and white matter (WM) for the whole brain and left and right hemisphere respectively in both datasets. **b**, The directionality of age-FD associations replicated in the validation dataset (dHCP: n = 782, UCI: n = 99)–independent of study site, scanner type, acquisition protocol, spatial resolution, age range, and parcellation. Error bars: 95% confidence intervals; center: correlation coefficients. Source data

First, the direction of age−FD effects closely replicated in an independent validation cohort from the University of California, Irvine (UCI, *n* = 99 newborns; Extended Data Fig. 5)^33,34^.

**Extended Data Fig. 6:**
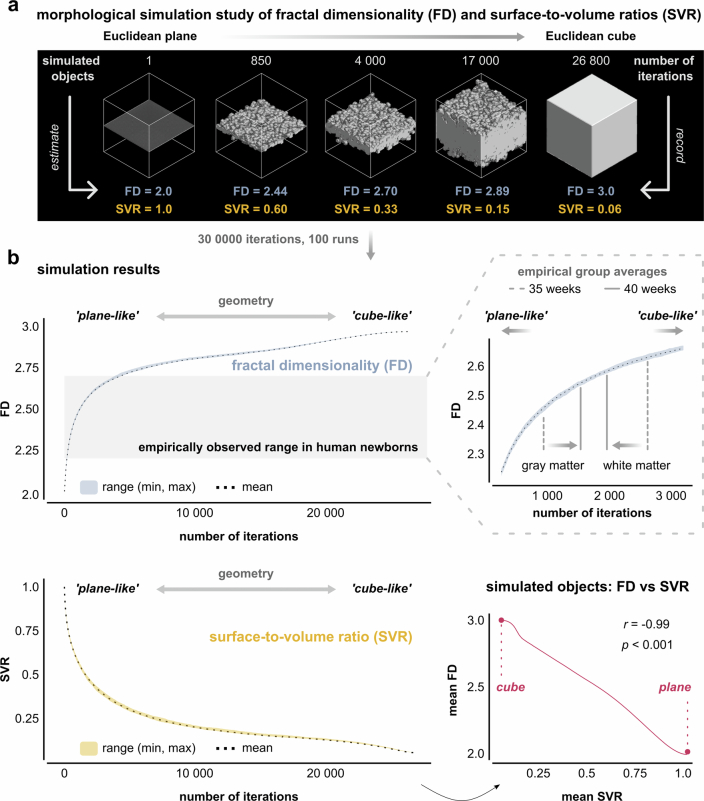
Morphological simulation study of fractal dimensionality (FD) and surface-to-volume voxel ratios (SVR). **a**, Illustration of a simulation run. The simulation starts from a binary matrix of 0s (100x100x100 voxels) filled with a plane of 1s (100x100x1) as the initial object (left panel). In every iteration, the FD and SVR of the simulated object are estimated as in the empirical data (see Methods). Subsequently, a surface voxel of the object is chosen at random and defined as the center of a 5x5x5 cube which is set to 1. Thus, the simulated object increasingly fills more of the embedding space, as it transforms from a Euclidean plane (theoretical FD = 2) into a fully filled cube (theoretical FD = 3, right panel). The simulation ran 100 times with 30,000 iterations each, and all runs arrived at the cube. **b**, Results of the simulation study. The upper row shows the FD of the simulated objects as they transition from a ‘plane-like’ to a ‘cube-like’ geometry, where the gray tile marks the numerical range observed empirically in neonatal brains. The inset on the right zooms in on this range. The lines mark the empirical group averages (dHCP data) for cortical gray matter and global white matter at 35 and 40 weeks, respectively. The simulation thus suggests that white matter (WM) starts out as a more ‘cube-like’ geometry in younger infants and develops into a more ‘plane-like’ geometry towards term maturity, while the opposite is true for cortical gray matter (GM). Furthermore, the simulation suggests that the geometric properties of GM and WM converge towards term maturity, reflected in a numerical convergence of GM- and WM-FD. The lower row shows the corresponding SVR of the simulated objects (left). The simulation suggests a strong inverse correlation between SVR and FD (right; two-tailed product-moment correlation test, *P* ≈ 0 within machine precision). These theoretical results from the simulation are tested empirically in Extended Data Figs. 7 and 8. Source data

Second, we conducted a morphological simulation study in which we gradually transformed a Euclidean plane (theoretical FD = 2) into a fully filled cube (theoretical FD = 3), illustrating how FD maps the geometric continuum between the idealized dimensions of Euclid (Extended Data Fig. 6). Additionally, this simulation showed a strong inverse relationship between the FD of the simulated objects and their surface-to-volume voxel ratios (SVRs), offering a geometric interpretation of FD as an index of how ‘space-filling’ an object is with regard to the embedding space.

**Extended Data Fig. 7:**
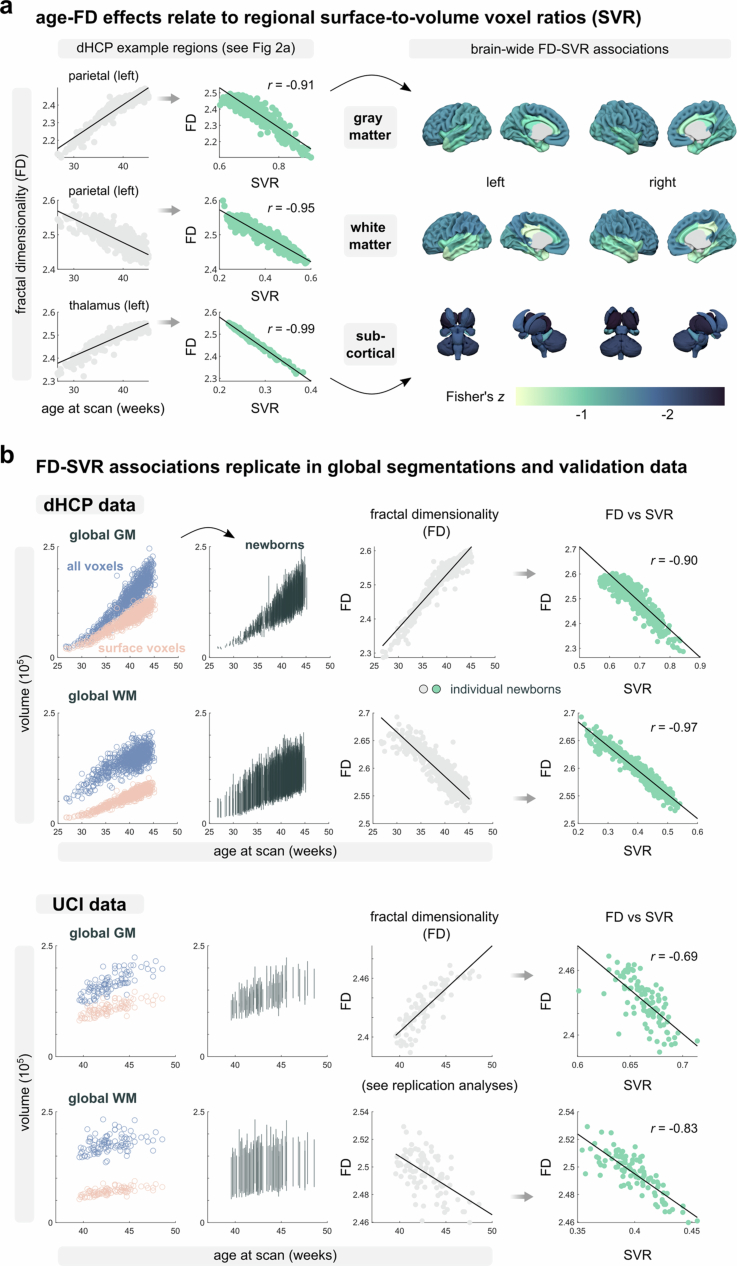
The theoretical relationship between fractal dimensionality (FD) and surface-to-volume voxel ratios (SVR) replicates in empirical data. **a**, Relating fractal dimensionality (FD) to surface-to-volume voxel ratios (SVR) in the parcellated brain data from the dHCP. The morphological simulation study (Extended Data Fig. 6) showed a principled inverse relationship between FD and SVR in simulated objects. The left panels show this relationship empirically for the three exemplary regions in main Fig. 2a. The right panel displays the brain-wide associations, showing that the inverse FD-SVR relationship is observed across all brain regions. Correlation values are z-transformed for visualization. **b**, The inverse relationship between FD and SVR replicates in global tissue segmentations (that is, independent of regional parcellation) and in the external validation data from the University of California, Irvine (UCI); see Extended Data Fig. 5 for details on this cohort.

Third, this theoretical association between FD and SVR in simulated objects was closely corroborated in the empirical brain data, in both the main cohort (dHCP) and the replication cohort (UCI) (Extended Data Fig. 7).

**Extended Data Fig. 8:**
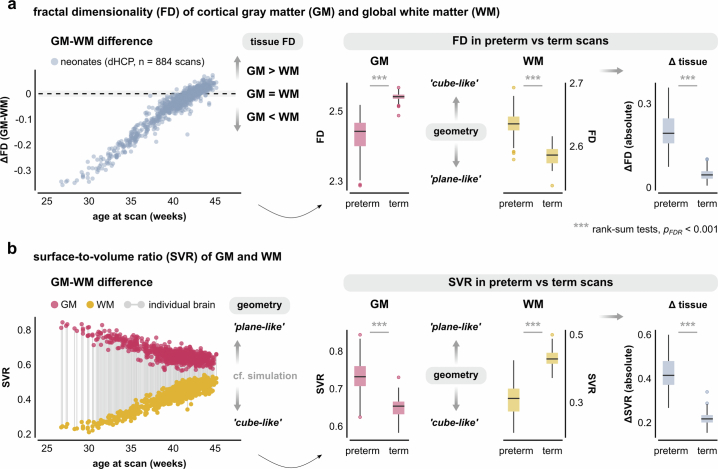
Biological inferences from morphological simulation study. **a**, Empirical findings for fractal dimensionality (FD) in the dHCP data (n = 884 scans). The simulation study (Extended Data Fig. 6) suggested that FD values of cortical gray matter (GM) and global white matter (WM) in individual brains should numerically converge towards term maturity. The left panel shows the FD difference between GM and WM in individual infants and confirms this development, with a GM-WM equilibrium point around 40-42 weeks. Furthermore, the simulation study revealed that the spatial properties of an object can be located on a continuous spectrum between a ‘plane-like’ geometry (FD towards 2) and a ‘cube-like’ geometry (FD towards 3). Accordingly, we compared scans in the preterm window to term-window scans (two-sided rank-sum tests with FDR adjustment) and found that GM develops from a more ‘plane-like’ to a more ‘cube-like’ geometry (middle-left; *P*_*FDR*_ = 4.2*10^−45^), while the opposite was true for WM (middle-right; *P*_*FDR*_ = 6.8*10^−43^). Consequently, the absolute GM-WM difference in FD was significantly lower in more mature brains (right panel; *P*_*FDR*_ = 4.2*10^−45^) **b**, Empirical findings for surface-to-volume voxel ratios (SVRs) in the dHCP data (n = 884 scans). The simulation study showed a strong inverse relationship between FD and SVR. Accordingly, the GM-WM convergence of FD values should be reflected by a similar convergence of SVR in more mature brains. The left panel confirms this development in individual brains. Similarly, the preterm vs term comparisons (two-sided rank-sum tests with FDR adjustment) again suggested that GM develops from a ‘plane-like’ (SVR towards 1) to a ‘cube-like’ geometry (SVR towards 0; middle-left; *P*_*FDR*_ = 1.7*10^−39^), while the opposite was observed for WM (middle-right; *P*_*FDR*_ = 3.5*10^−44^). As for FD, the absolute GM-WM difference in SVR was significantly lower in more mature brains (right panel; *P*_*FDR*_ = 4.2*10^−45^). Boxes in panels (**a**) and (**b**) display the interquartile range (IQR; lower hinge: 25^th^ percentile; upper hinge: 75^th^ percentile; center line: median), and whiskers cover the furthest data points within 1.5*IQR.

Fourth, we tested the geometric interpretation of the simulation study in the empirical data, which suggested that GM develops from a more ‘plane-like’ geometry in younger infants (FD toward 2, SVR toward maximum, less space-filling) to a more ‘cube-like’ geometry in older infants (FD toward 3, SVR toward minimum, more space-filling), whereas the opposite development was observed for WM geometry (Extended Data Fig. 8).

**Extended Data Fig. 9:**
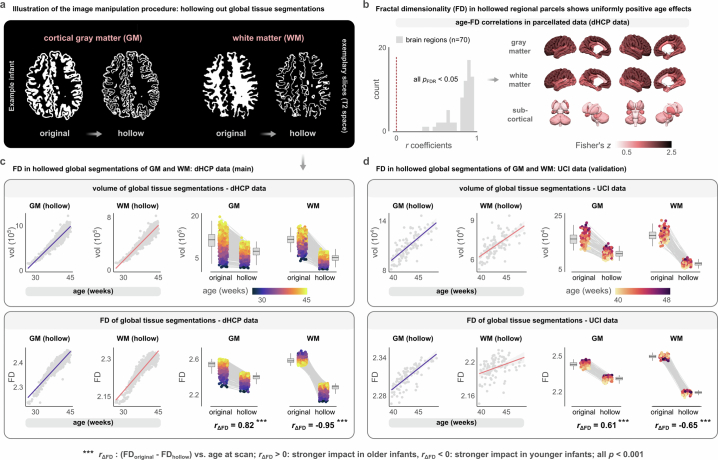
Hollowing out tissue segmentations. **a**, Illustration of the image manipulation in exemplary slices from a dHCP infant. The original tissue segmentations are hollowed out, such that inside voxels of the segmentation mask are removed. The hollowed-out masks for cortical gray matter (GM) and global white matter (WM) are more similar to each other, as the GM-WM boundary closely follows the cortical ribbon. **b**, Regional segmentations (dHCP data). Computing FD from the hollowed-out regional segmentations results in uniformly positive age associations across all brain areas. The direction of age correlations thus remains unchanged for all cortical GM and subcortical areas, whereas it changes uniquely for those WM regions that show inverse age associations when the original segmentations are assessed (Fig. 2; Extended Data Figs. 1 and 5). **c**, Global tissue segmentations (dHCP data, n = 782). Effects of hollowing on volume (upper row) and FD (lower row) of global tissue segmentations. As voxels are removed by the hollowing procedure, volumes are naturally lower for both GM and WM. Notably, hollowed global segmentations yield uniformly positive age-FD associations, as for parcellated data. Additionally, FD values in hollowed segmentations are universally lower than in the original segmentations, closely corroborating previous studies^17^. With reference to the simulation results (Extended Data Fig. 6), the hollowing procedure can thus be interpreted to impose a more ‘plane-like’ geometry on the segmentations. Consequently, the effect of hollowing on FD is stronger in WM than in GM (cf. slopes in lower-right plots) because the latter already shows a more ‘plane-like’ behavior to begin with (Extended Data Figs. 6b and 8a). Similarly, the effect of hollowing was stronger in *older* infants for GM (because GM develops from a ‘plane-like’ to a ‘cube-like’ geometry), but stronger in *younger* infants for WM (because WM develops from a ‘cube-like’ to a ‘plane-like’ geometry; Extended Data Figs. 6b and 8a), as quantified by the *r*_ΔFD_ values (two-sided product-moment correlation test: GM: *P* = 2.6*10^−188^; WM: *P* ≈ 0 within machine precision). **d**, Global tissue segmentations (UCI data, n = 99). These effects of hollowing were closely corroborated in the UCI validation data (two-sided product-moment correlation test: *r*_ΔFD_ GM: *P* = 2.9*10^−11^; *r*_ΔFD_ WM: *P* = 3.7*10^−13^), albeit with smaller effect sizes (two-sided Fisher’s *z*-test: GM: Δr=0.21 [0.09,0.35], z = 4.0, *P* = 5.2*10^−5^; WM: Δr = -0.29 [-0.43,-0.19], z = -9.3, *P* ≈ 0 within machine precision). Boxes in panels (**c**) and (**d**) display the interquartile range (IQR; lower hinge: 25^th^ percentile; upper hinge: 75^th^ percentile; center line: median), and whiskers cover the furthest data points within 1.5*IQR. Source data

Fifth, we thus hypothesized that the inverse age−FD in WM could be flipped by artificially imposing a more ‘plane-like’ geometry on the WM segmentations through a hollowing procedure (and thereby making them more similar to cortical GM). This effect was indeed observed and again replicated in the validation data (Extended Data Fig. 9).

**Extended Data Fig. 10:**
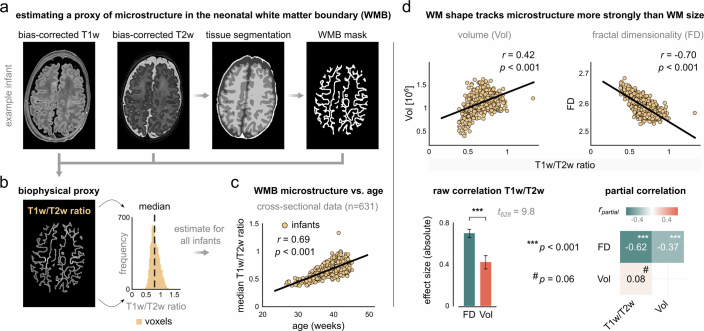
Microstructural developments of neonatal white matter (WM) relate significantly more strongly to fractal dimensionality (FD) than to volume. **a**, Illustration of the data used to estimate the ratio of T1-weighted and T2-weighted data (T1w/T2w) as a biophysical proxy of microstructure in the neonatal white matter boundary (WMB). Representative slices of bias-corrected T1-weighted data, bias-corrected T2-weighted data, and the tissue segmentation, all in T2w space, as provided by the dHCP. The mask of the white matter boundary (WMB) is computed by finding the WM voxels bordering on cortical gray matter and sampling inside the WM mask to 1 mm depth. Data from an exemplary neonate. **b**, A microstructural proxy of the neonatal WMB is estimated as the voxel-wise ratio of T1-weighted and T2-weighted data^15,79,80^ and illustrated here for the slices from panel (**a**). Voxel-wise data of each infant were aggregated as the median T1w/T2w ratio over all WMB voxels (right inset). **c**, WMB T1w/T2w ratio is positively related to infant age (data available for n = 631 neonates; two-tailed product-moment correlation test, *P* = 7.7*10^−91^). **d**, Association between the microstructural proxy (T1w/T2w) and WM volume (Vol; upper left; *P* = 1.1*10^−28^) and WM fractal dimensionality (FD; upper right; *P* = 2.9*10^−94^; two-tailed product-moment correlation tests, n = 631). The T1w/T2w-FD association is significantly stronger than the T1w/T2w-volume relationship (lower left, error bars: 95% confidence intervals; center: absolute correlation coefficient; two-sided Williams’ test, *P* ≈ 0 within machine precision). Partial product-moment correlation tests between all three variables showed that the T1w/T2w-volume relationship vanishes when controlling for FD, whereas the T1w/T2w-FD relationship persists when controlling for volume (lower right; FD vs. T1w/T2w, *P* = 4.8*10^−68^; FD vs. Vol, *P* = 2.3*10^−22^; Vol vs. T1w/T2w, *P* = 0.057). Source data

Finally, a further validation study showed that the T1w/T2w ratio as a biophysical proxy of WM microstructure related significantly more strongly to WM−FD than to volume (Extended Data Fig. 10).

### Brain shape outperforms brain size in predicting infant age

**Fig. 5:**
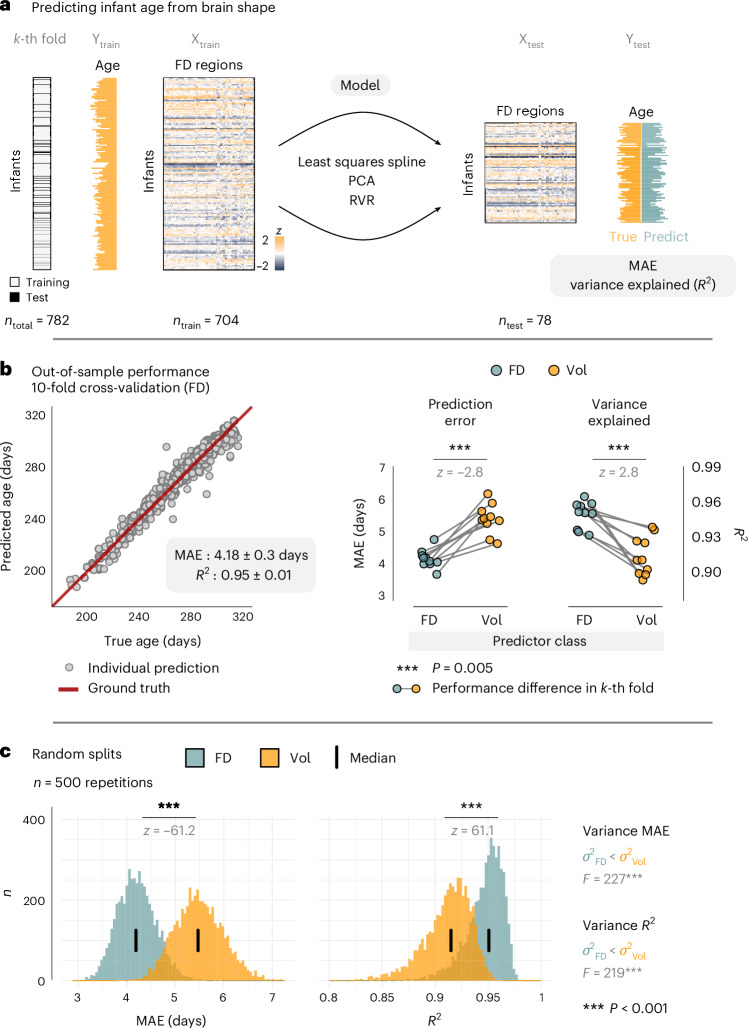
Brain shape outperforms brain size in predicting infant age. **a**, Schematic of the age prediction pipeline, resting on a combination of least squares splines, principal component analysis (PCA) and relevance vector regression (RVR). Model performance in unseen data was evaluated by mean absolute error (MAE) of age prediction in days and variance explained in test data (*R*^2^), employing a 10-fold cross-validation scheme. **b**, Out-of-sample performance of predicting infant age from FD (left) and fold-wise comparisons between FD-based and volume-based age prediction (right) using two-sided signed-rank tests (*n* = 10 folds; *P* = 0.005 for both MAE and *R*^2^). **c**, Repetitions of the cross-validation procedure over random data splits to estimate the distribution of performance metrics with respect to variance and location. Differences in location between FD-based and volume-based prediction were assessed with two-sided signed-rank tests (*P* ≈ 0 within machine precision for both MAE and *R*^2^); differences in variance were assessed with Levene’s test (MAE: *P* = 9.4 × 10^−51^; *R*^2^: *P* = 5.6 × 10^−49^). Vol, volume. Source data

Given these inferential age−FD effects, we next asked how closely infant age could be predicted from brain shape in unseen data. To this end, we employed a supervised age prediction scheme, resting on a combination of least squares splines, dimensionality reduction and relevance vector regression^25,35^. Herein, FD values constituted the predictor matrix, and the quality of age prediction was assessed as the mean absolute prediction error (MAE) in days and variance explained (*R*^2^) in unseen data, evaluated using a 10-fold cross-validation scheme (Fig. 5a).

Out-of-sample performance of age prediction yielded high accuracy, with a mean prediction error of 4.2 ± 0.3 days and a substantial amount of variance explained in the test data (*R*^2^ = 0.95 ± 0.01) (Fig. 5b). Furthermore, shape-based age prediction with FD significantly outperformed size-based age prediction with volume—both in terms of lower prediction errors (*z* = −2.8, *P* = 0.005) and more variance explained over individual folds (*z* = 2.8, *P* = 0.005) (Fig. 5b). Notably, volume-based prediction tended to overestimate age in very young infants (Extended Data Fig. 3b), which was not observed with FD.

Moreover, we repeated the cross-validation procedure over *n* = 500 random splits of the dataset into the 10 respective folds (that is, 5,000 unique test sets) and evaluated the resulting distributions of the performance metrics for differences in location and variance. This approach corroborated the superior performance of FD in terms of both prediction errors (*z* = −61.2, *P* < 0.001) and variance explained (*z* = 61.1, *P* < 0.001) and yielded significantly lower variance of the performance metrics (MAE: *F* = 277, *P* < 0.001; *R*^2^: *F* = 219, *P* < 0.001), suggesting that age prediction from brain shape generalized substantially better over random fluctuations in the data (Fig. 5c).

Finally, we conducted three additional control analyses. First, age prediction from FD and volume together performed on par with age prediction from FD alone (MAE: 4.1 ± 0.4 days, ΔMAE = 0.15 ± 0.25 days versus FD; *R*^2^ = 0.95 ± 0.02, Δ*R*^2^ = 0.5 ± 0.6% versus FD). Second, the superior performance of age prediction from FD was confirmed in two alternative control models of lower model complexity (multiple linear regression and support vector regression), with virtually identical results (Supplementary Fig. 1). Third, FD predicted not only age at scan but also age at birth with high accuracy, even when infants were scanned up to 1 month after birth (Supplementary Fig. 2a). Similarly, FD significantly outperformed volume in a supervised binary classification approach of preterm versus term birth from term window scans (Supplementary Fig. 2b).

### Brain shape detects signatures of prematurity that are not captured by brain size

**Fig. 6:**
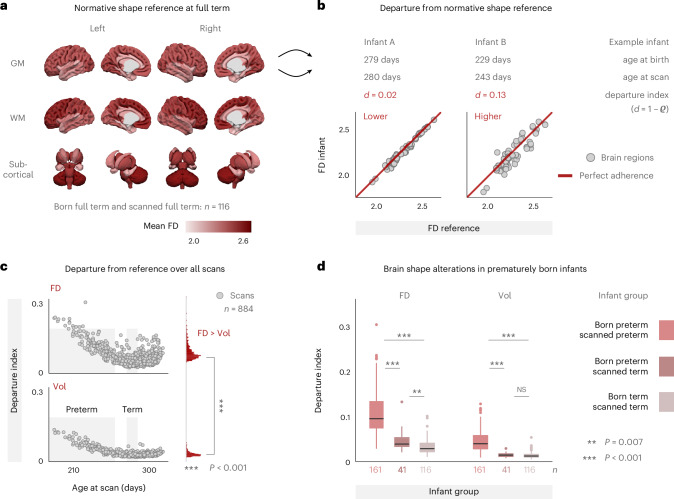
Brain shape detects signatures of prematurity that are not captured by brain size. **a**, Normative reference derived as the mean FD per region over all infants who were both born and scanned at full term (*n* = 116, defined as 39 0/7 weeks to 40 6/7 weeks, following the ACOG criteria^31^). Regions are based on the modified ALBERT parcellation^12,13^ (Methods). **b**, Quantifying the departure from this reference with a departure index ‘*d*’, computed as the spatial rank correlation distance between each infant’s individual values and the reference values from **a**. Illustration for two infants with lower departure from reference (left; born term, scanned term) and higher departure from reference (right; born preterm, scanned preterm). Reference brain size was computed in analogy using regional volumes. **c**, Departure from reference over all *n* = 884 scans in the dataset for brain shape (top) and brain size (bottom). The shaded areas display the ACOG definitions of preterm age (<37 0/7 weeks = 259 days) and term age (273−286 days). Note the local minimum of both scatter clouds around the term window. Departure indices were significantly higher for FD than for volume (two-sided rank-sum test, *P* = 8.2 × 10^−175^). **d**, Departure from reference for three infant groups: (1) born preterm and scanned preterm (*n* = 161), (2) born preterm and scanned term (*n* = 41) and (3) born term and scanned term (*n* = 116). Boxes display the interquartile range (IQR; lower hinge: 25th percentile; upper hinge: 75th percentile; center line: median), and whiskers cover the furthest data points within 1.5× IQR. Kruskal−Wallis omnibus tests yielded significant results for both FD (*χ*^2^_2_ = 197.2, *P* = 1.5 × 10^−43^) and volume (*χ*^2^_2_ = 194.6, *P* = 5.5 × 10^−43^). Pairwise comparisons correspond to Dunn’s tests with FDR adjustment (FD: term−term versus preterm−term: *P*_FDR_ = 0.007; term−term versus preterm−preterm: *P*_FDR_ = 1.8 × 10^−42^; preterm−term versus preterm−preterm: *P*_FDR_ = 2.0 × 10^−12^; Vol: term−term versus preterm−term: *P*_FDR_ = 0.205; term−term versus preterm−preterm: *P*_FDR_ = 2.6 × 10^−39^; preterm−term versus preterm−preterm: *P*_FDR_ = 8.1 × 10^−17^). NS, not significant; Vol, volume.

Next, we asked what normative brain shape is expected in infants of full-term maturity. To address this question, we estimated a full-term FD reference and quantified how much the brains of individual infants departed from this reference. For each brain region, we thus computed the average FD values over those infants who were both born and scanned within the full-term window, which applied to *n* = 116 neonates (Fig. 6a; size reference calculated in analogy from volumes). This approach allowed us to compute a whole-brain ‘departure index’ as the spatial correlation distance between these reference values and an infant’s individual values. Figure 6b illustrates this procedure for one infant who was born and scanned at full term and shows low departure from reference (left) and another infant who was born and scanned preterm and shows higher departure from the normative reference (right). Furthermore, the distribution of departure indices over all scans (Fig. 6c) revealed that (1) departure from normative shape is significantly stronger than departure from normative size (rank-sum test: *z* = 28.2, *P* < 0.001); (2) departure indices across individual scans are significantly more variable for shape than for size (*F*-test: *F*_883,883_ = 5.6, *P*< 0.001); and (3) both distributions show a local minimum around term age at scan, which is expected because this is the age window on which the respective references were defined. These distributions subsequently allowed for explicit comparisons among three infant groups: (1) those born preterm and scanned preterm (preterm−preterm, *n* = 161); (2) those born preterm but later scanned at term-equivalent age (preterm−term, *n* = 41); and (3) those born at term and scanned at term (that is, the reference group; term−term, *n* = 116). Consistent with the previously observed age effects, group 1 (preterm−preterm) showed significantly higher departure from the normative reference than both group 2 (preterm−term) and group 3 (term−term), and this held true for both FD and volume (Fig. 6d). By contrast, the comparison between group 2 (preterm−term) and group 3 (term−term) was significant only for FD but not for volume (Fig. 6d), showing that brain shape captured signatures of premature birth even when those infants were later scanned in the full-term age window, whereas such signatures of prematurity were not detected with brain size.

As an exploratory follow-up, we furthermore estimated which brain regions are most implicated in these shape differences by relating the regional FD values of individual infants to the distribution of FD values in the term−term reference (Supplementary Fig. 3a). Therein, we found strong deviations from reference in the preterm−preterm group across virtually all brain regions (Supplementary Fig. 3b), corroborating both the strength and direction of the previously observed age effects. Interestingly, however, the preterm−term group showed a differentiated pattern of shape deviations, in which some brain regions were not significantly different from the term−term reference (for example, occipital cortex), other areas were still ‘lagging behind’ the reference (for example, brainstem) and yet other areas showed an ‘overshoot’ of FD values compared to the reference (for example, frontal cortex) (Supplementary Fig. 3c).

### Brain shape reflects genetic information

Next, we moved beyond group-level age effects and studied the relationship between genetic factors and brain shape on the level of individual newborns.

**Fig. 7:**
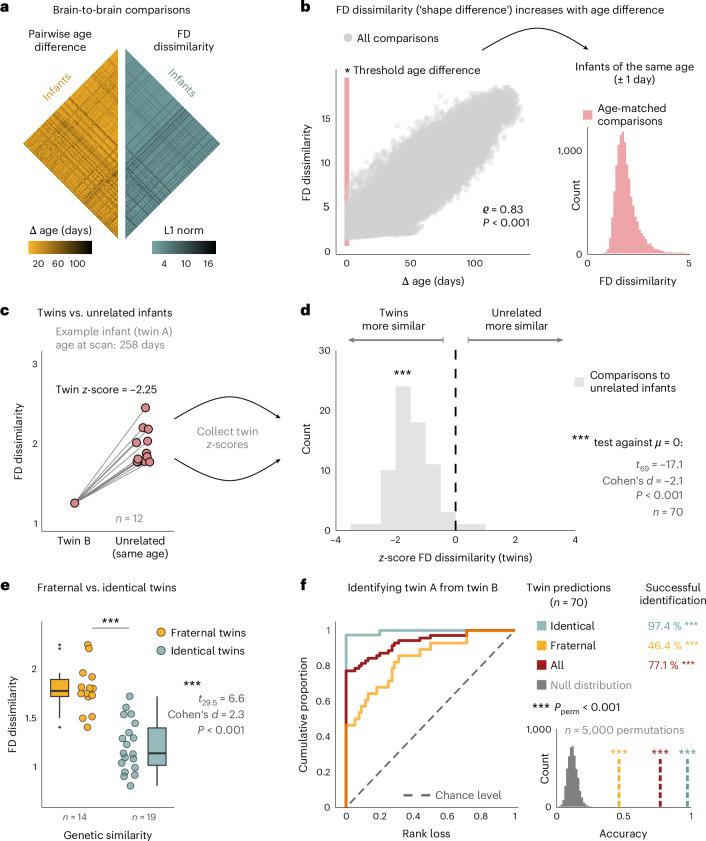
Brain shape reflects genetic similarity among newborns and enables the identification of one twin from the brain of the other twin. **a**, Pairwise brain-to-brain comparisons across the dHCP dataset. The matrices display the age differences of the compared infants (left) and the overall ‘shape difference’ of their brains (right), computed as the dissimilarity of their FD values across all brain regions. **b**, Correlation between age differences and FD dissimilarity for all brain-to-brain comparisons (left; Spearman’s rank correlation, *P* ≈ 0 within machine precision). Age-matched dissimilarity distribution after thresholding the age difference to ±1 day (right). **c**, Dissimilarity scores between an exemplary infant and its twin sibling (left) and all unrelated infants of the same age (right). The dissimilarity of the twin sibling was *z*-scored with regard to all age matches and collected for each twin-to-unrelated comparison (70 comparisons across *n* = 470 infants). **d**, Distribution of twin dissimilarities from **c** over all twin-to-unrelated comparisons. One-sample *t*-test against zero (two-sided, *P* = 1.4 × 10^−26^). **e**, Dissimilarity scores across fraternal and identical twin pairs (Welch’s *t*-test, *P* = 3.2 × 10^−7^). Boxes display the interquartile range (IQR; lower hinge: 25th percentile; upper hinge: 75th percentile; center line: median), and whiskers cover the furthest data points within 1.5× IQR. **f**, Predicting twin siblings from brain shape. For each twin-to-unrelated comparison (see **c**), the infant with the lowest-ranking dissimilarity was predicted to be the target twin. The ROC-like curve shows the proportion of infants over increasing rank loss (0: correct identification; 1: all unrelated infants more similar than target twin). The null distribution of twin predictions was estimated by randomly permuting the rank structure and recording the correct twin identifications that happen by chance. *P* values of the permutation test are given by the number of permuted accuracies that surpass the empirically observed accuracy, divided by the number of permutations (*n* = 5,000; all zero here). ROC, receiver operating characteristic. Source data

To this end, we computed the pairwise age differences for all infant-to-infant comparisons in the dataset and measured the ’shape difference’ of their brains as the dissimilarity of their whole-brain FD profiles (Fig. 7a). As expected from the group-level effects, the shape difference between any two infants strongly increased with the age difference between them (*ρ* = 0.83, *P* < 0.001; Fig. 7b). However, the granularity of individual brain-to-brain comparisons allowed us to threshold the pairwise age differences to obtain only those comparisons in which both infants were within 1 day of age at the time of scanning. The inset of Fig. 7b shows that, even within this subset of age-matched comparisons, there is considerable variance in the FD dissimilarity of individual brains. Notably, however, these shape differences are not attributable to age because the respective infants were the same age at the time of scanning, allowing us to evaluate if sharing genetic information—beyond sharing the same age—would be linked to a higher similarity in brain shape.

To test this idea, we first compared the brains of twin siblings to all matched infants who were the same age as these twins but biologically unrelated to them. Figure 7c illustrates the resulting dissimilarity distribution for one of the 35 twin pairs for whom unrelated age matches were available. Here, the difference between the exemplary infant and its twin sibling was substantially lower than the difference to any of the unrelated children, such that the two twin brains were the most alike in shape. Critically, this observation generalized over all twin-to-unrelated comparisons—brain shapes of twin siblings were generally more similar to each other than to the brains of unrelated infants, with large effect size (one sample *t*-test: *t*_69_ = −17.1, *P* < 0.001, Cohen’s *d* = −2.1; Fig. 7d).

Consequently, we performed two additional analyses to test the idea that similarity in brain shape may reflect similarity in genetic information.

First, we stratified the dissimilarity scores by the sex of the compared infants (Supplementary Fig. 4). This revealed that infants of the same sex exhibit significantly more similar brain shapes than infants of different sexes, and this was true in both twin siblings and biologically unrelated infants. Interestingly, for infants of the same sex, brain shapes tended to be even more similar when both newborns were female compared to when both newborns were male (*z* = −6.2, *P*_FDR_ < 0.001 for unrelated, tendency in twins), suggesting an additional effect of homologous sex chromosomes that share the same genes (that is, an XX karyotype in both infants) compared to heterologous sex chromosomes (that is, an XY karyotype) that do not.

Second, we hypothesized that, even among twins, sharing more genetic information would be reflected by yet more similar brain shapes. Accordingly, we stratified twins into dizygotic siblings (that is, fraternal twins with approximately 50% shared genes) and monozygotic siblings (that is, identical twins with approximately 100% shared genes) and indeed observed that brain shapes are significantly more similar in identical twins than in fraternal twins (*t*_29.5_ = 6.6, *P*< 0.001, Cohen’s *d* = 2.3; Fig. 7e).

Notably, analogous control analyses with volume showed that genetically related infants exhibit stronger similarity in brain shape than in brain size (Supplementary Fig. 5).

### Identifying the brain of one twin from the brain of the other twin

Given these findings, we lastly asked if brain shape would enable the identification of twin siblings among age-matched unrelated infants (Fig. 7f). This approach pertains to the idea of ‘connectome fingerprinting’^36^, in which the unique variability of brain activity signatures (‘fingerprints’) enables the identification of single individuals with high accuracy. Notably, however, here we do not aim to identify the same individual but, rather, the individualʼs twin sibling. To this end, the dissimilarity scores of individual twin-to-unrelated comparisons were ranked, and the infant with the lowest-ranking shape difference was predicted to be the other twin. In the example of Fig. 7c, the twin sibling was thus correctly identified but not so in the analogous analysis with volume (Supplementary Fig. 5a). To assess the predictive power of this approach, we computed (1) the ‘rank loss’ over individual predictions, defined as the proportion of unrelated infants whose brain shapes were more similar to the target infant than its twin (that is, rank loss = 0: correct identification; rank loss = 1: all unrelated more similar than twin; Fig. 7f, left); (2) the accuracy of twin predictions as the proportion of correct identifications; and (3) the null distribution of correct twin identifications that happen by chance. The latter was estimated by randomly permuting the ranks within individual predictions, yielding the permuted *P* value (*P*_perm_) on the prediction accuracy as the proportion of randomly obtained accuracies that surpass the empirically observed value (lower-right inset). On average, approximately 11% of twin identifications were thus expected to happen by chance.

Critically, brain shape correctly identified the target twin in 77.1% over all predictions (*P*_perm_ < 0.001; Fig. 7f). Notably, however, predictive power again mirrored the effect of genetic similarity on shape similarity: whereas the accuracy of identifying fraternal twins was considerably lower (46.4%), if still far from chance (*P*_perm_ < 0.001), prediction accuracy was near perfect in the case of identical twins (97.4%, *P*_perm_ < 0.001; Fig. 7f).

Here again, analogous analyses with volume showed that predictive power of brain size was markedly lower, resulting in a consistent 25−30% drop in identification accuracy (Supplementary Fig. 6).

**Fig. 8:**
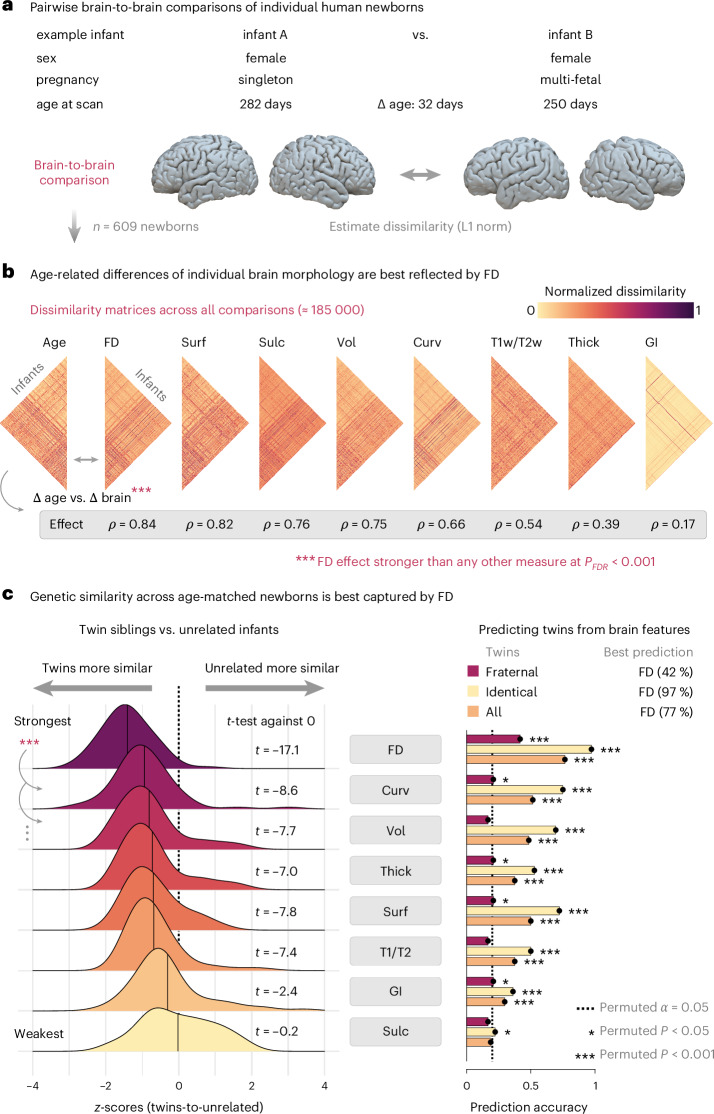
FD outperforms earlier measures in capturing the morphological variability of individual brains, detecting genetic similarity and predicting which newborns are twin siblings. **a**, Assessing the morphological variability of individual brains through pairwise brain-to-brain comparisons, as in Fig. 7 and illustrated here for two example neonates. The age difference between any two newborns is related to the morphological dissimilarity of their brains (as in Fig. 7b), computed as the L1 norm over regions for each of eight brain measures: cortical thickness (Thick), curvature (Curv), gyrification index (GI), sulcation (Sulc), surface area (Surf), T1w/T2w ratio (T1/T2), volume (Vol) and FD. Complete data were available for a subset of *n* = 609 newborns from the dHCP. **b**, The resulting dissimilarity matrices across all brain-to-brain comparisons (*n* = 185,136). The age-related dissimilarity of any two brains is captured most strongly by FD (descending effect strength from left to right). Correlations were statistically compared with a permutation test, showing that the age effect was significantly stronger for FD compared to all other metrics (*P*_FDR_< 0.001). **c**, Genetic analyses in age-matched newborns (compare to Fig. 7d–f). The left panel shows that FD most strongly discriminates between twin siblings and unrelated newborns of the same age (64 twins-to-unrelated comparisons across a total of *n* = 423 infants), and this effect was significant at *P*_FDR_ < 0.001 compared to all other measures (two-sided paired *t*-tests versus FD: Curv: Cohen’s *d* = −0.49, *P*_FDR_ = 2.1 × 10^−4^; Vol: Cohen’s *d* = −1.02, *P*_FDR_ = 5.1 × 10^−11^; Thick: Cohen’s *d* = −0.78, *P*_FDR_ = 6.0 × 10^−8^; Surf: Cohen’s *d* = −0.96, *P*_FDR_ = 2.4 × 10^−10^; T1w/T2w: Cohen’s *d* = −0.73, *P*_FDR_ = 2.6 × 10^−7^; GI: Cohen’s *d* = −1.03, *P*_FDR_ = 4.2 × 10^−11^; Sulc: Cohen’s *d* = −1.16, *P*_FDR_ = 1.4 × 10^−12^). The right panel shows the twin prediction accuracies over all twins and for fraternal and identical twins separately (60 twin predictions across a total of *n* = 401 infants, assessed by permutation tests; compare to Fig. 7f). For exact *P* values not listed here, see the Supplementary Information. Source data

Finally, we repeated the core analyses of brain-to-brain comparisons in the subset of newborns for whom surface-derived brain measures were available (*n* = 609), including cortical thickness, curvature, gyrification, sulcation, surface area and T1w/T2w ratio (Fig. 8a). Therein, we found that FD (1) significantly outperformed all other metrics in capturing age-related differences of individual brain morphology (Fig. 8b; *P*_FDR_ < 0.001 for all comparisons); (2) was the most sensitive in discriminating genetically related from age-matched unrelated infants (Fig. 8c, left; all *P*_FDR_ < 0.001); and (3) showed the highest power in predicting which newborns are twin siblings (Fig. 8c, right), both overall (accuracy 77%) and separately for fraternal twins (42%) and identical twins (97%) (*P*_perm_ < 0.001 for all).

## Discussion

These findings show that the early-life formation of brain shape represents a fundamental maturational process in human brain development.

To study these shape developments, we analyze structural MRI data from the dHCP, one of the largest datasets of human newborns ever collected^11^. Therein, we describe brain shape with fractal dimensionality, a geometric measure of structural complexity that complemented and systematically outperformed purely size-based accounts of neonatal brain development. Specifically, we found that brain shape (1) strongly reflects infant maturity beyond size differences, both cross-sectionally and longitudinally; (2) consistently outperforms brain size in predicting infant age in unseen data, with high accuracy (mean error approximately 4 days); (3) detects signatures of prematurity that are not captured by brain size; (4) is consistently more sensitive to genetic similarity among newborns, assessed by comparing infant sex, related versus unrelated infants and fraternal versus identical twins; and (5) enables the identification of one twin from the brain of the other twin with high accuracy (approximately 77% overall, 97% in identical twins), again outperforming twin predictions from brain size. Additionally, FD was systematically better at capturing infant age, the morphological variability of individual brains and genetic information when compared to common surface-derived measures, including cortical thickness, curvature, gyrification, sulcation, surface area and the T1w/T2w ratio.

Below, we turn to the implications of these findings, which advance our understanding of early-life brain development along six key directions.

First, brain shape is inextricably linked to infant age, closely capturing inter-individual and intra-individual differences in infant maturity. Therein, age−FD associations showed a highly consistent spatial pattern, which was observed both cross-sectionally and longitudinally and in which more mature brains are characterized by higher GM−FD and lower WM−FD. Notably, these effects replicated in an external validation cohort and closely reflected a biophysical proxy of microstructural WM development. To derive a geometric interpretation of these effects, we implemented a morphological simulation study that produced objects of dimension $2\le {\rm{FD}}\ge 3$, spanning a continuum between a more ‘plane-like’ geometry (FD toward 2) and a more ‘cube-like’ geometry (FD toward 3). The empirically observed age effects thus indicate that GM develops from a more ‘plane-like’ geometry in younger infants to a more ‘cube-like’ geometry in more mature brains, whereas the opposite trend was observed for WM.

Of course, this is not to say that either tissue compartment looks like a plane or a cube visually. Rather, it is their geometric properties (and specifically their spatial scaling exponents) that exhibit a more plane-like or cube-like behavior. An intuitive interpretation of this can be invoked by the notion of how ‘space-filling’ an object is with regard to the embedding space, where the latter here corresponds to the three-dimensional matrix representing the MRI. Consequently, our findings suggest that cortical GM develops from a less space-filling to a more space-filling structure, whereas WM shows the opposite development. Notably, however, this relationship refers to the object’s dimension, which is independent of its absolute size (Methods). That is, even though both GM and WM are naturally larger in older infants due to brain growth, FD quantifies their space-filling properties relative to the embedding space.

Biologically, FD should thus be interpreted as a developmental marker of early-life brain maturity, which is rooted in geometry rather than size. Accordingly, a geometric account of our findings is that the cerebral cortex starts out as a relatively smooth sheet (FD closer to 2) and becomes gradually more space-filling with increasing convolution. By contrast, WM starts out as more of a solid block (FD closer to 3) and becomes less space-filling, possibly due to increasing sulcal indentation. Incidentally, in the oldest infants, GM−FD ultimately surpassed WM−FD numerically, which is also observed in adult brains^16^.

Second, this spatial pattern was paralleled by temporal differences in the developmental trajectories, in which cortical GM showed the most rapid change over time, whereas WM showed a more protracted evolution. These findings in neonates are consistent with early work on brain growth trajectories over the first 2 years of life, which reported slower WM development compared to cortical GM^37^. Here, we observe similar temporal differences in brain shape formation and show that such tissue-specific dynamics are already present at birth, beyond volumetric growth^38^. Notably, these perinatal dynamics also converge with a recent account of normative brain growth over the larger lifespan^1^, which suggested that developmental trajectories are steeper for GM than for WM around birth.

Third, it is particularly worth focusing on the development of cortical complexity, which constituted some of the strongest effects throughout our study. In general, our results suggest that the dynamic complexity increases in the cortex are an expression of early-life cortical folding. This folding process accelerates markedly around 26 weeks of gestational age, when the brain begins a rapid change from a near-lissencephalic to a highly convoluted structure in utero^39–41^. Here, we show that this morphological development naturally extends into the neonatal period, where the increasing cortical convolution is reflected by a highly canonical increase in structural complexity. In this context, recent evidence from statistical physics suggests that cortical morphologies across a variety of primate species may be an approximation of an underlying archetypal fractal shape^42^. Given the shape developments observed here, the formation of cortical complexity may thus not only represent a key process in human brain development but may rather be the result of a more general, evolutionarily conserved mechanism of cortical expansion^43–45^, possibly related to latent scaling rules. Although the precise biomechanics of this process are still being unraveled, a differential tangential growth of the outer cortex is thought to represent one key mechanism for cortical folding^46–48^, which raises the exciting possibility that future work may be able to bridge these microscale accounts of cortical development and the macroscale shape phenotypes studied here.

Fourth, we show that age differences do not only explain differences in brain shape but that this relationship can be inverted to predict the age of an infant from the shape of its brain with high accuracy. Here again, brain shape significantly outperformed brain size, and this was consistently observed across performance metrics, data splits and three different prediction models. Notably, prediction accuracy was homogeneously high across the whole age range in the dataset, from very premature to well after term, suggesting that brain shape closely reflects infant maturity over all stages of neonatal development. In this context, recent work has applied geometric deep learning (GDL) to show that shape characteristics of the human cortex are predictive of a person’s sex and age over the larger lifespan^49^, and GDL has also been used for neonatal age prediction from cortical features in the dHCP^50,51^. Notably, shape-based age prediction in our study generally performed at least on par with these reports, further highlighting FD as a promising new neuroimaging phenotype. In this context, the ‘ground truth’ ages as used here are commonly determined from self-reports of the mother’s last menstrual period, which represents a potential source of uncertainty. Therefore, our findings raise the question if brain shape can also predict fetal age in utero and how this compares to early-life ultrasound.

Fifth, brain shape captured morphological signatures of premature birth that remained undetected by brain size. Specifically, even when preterm-born infants were subsequently scanned in the term-equivalent age window, their brain shapes still deviated significantly from a normative reference of term-born infants, whereas this was not the case for brain size. In this context, a recent study on cortical structure after preterm birth found prematurity-related alterations to be highly variable over individual neonates^52^. Notably, our modeling framework explicitly allows for such individual deviation patterns because the departure index is agnostic to the direction of deviations and the particular regions involved in them, yielding an individualized new normative approach.

Besides these spatial alterations, we observed differences in individual temporal trajectories, where the brains of more prematurely born infants showed a higher rate of change compared to term-born infants. Ultimately, longitudinal studies contrasting in utero versus ex utero development are necessary to understand if this effect represents the normal developmental dynamics or, possibly, an acceleration in response to premature birth. Here, exploratory analyses indeed suggested that some brain regions show a developmental ‘overshoot’ in preterm-born infants, but more work is needed to unravel such spatial specificity comprehensively.

In sum, brain shape reflected altered developmental trajectories of preterm-born infants already within the first few postnatal weeks. Although this is, to our knowledge, the earliest account of altered shape development after preterm birth, one previous study applied fractal analysis in infants at 12 months and found that prematurely born infants with intra-uterine growth restriction showed persistent reductions in GM complexity that were related to language and motor scores^53^. Moreover, recent work reported persistent reductions of cortical complexity at adult age in participants who had been born prematurely, which was related to the degree of prematurity and correlated with reduced cognitive performance in adulthood^54^. These findings not only align well with the shape alterations observed here in newborns but also suggest such changes to carry functional significance for neurocognitive development.

Importantly, about 11% of infants are born prematurely worldwide^30^, bearing an increased risk for early-life mortality^30,55^, later-life cognitive deficits^8^ and neuropsychiatric disorders^56^. Our findings thus call for long-term longitudinal efforts to assess the prognostic potential of FD and follow up neonates into infancy and adulthood when neurodevelopmental disorders become manifest.

Sixth, our study reveals a systematic link between brain shape and genetic information. Specifically, we found that (1) the brains of genetically related infants are more similar in shape than those of unrelated infants; (2) infants of the same sex show more similar brain shapes than infants of different sexes; (3) brain shapes are more similar in homologous than in heterologous sex chromosomes; and (4) brain shapes are more similar in identical twins (approximately 100% shared genes) than in fraternal twins (approximately 50% shared genes). Notably, all these comparisons were carried out in age-matched infants, such that these results are unlikely to be confounded by the strong age effects discussed above.

These findings complement the fast-growing literature linking neuroimaging phenotypes to genetic factors in human brain development^2,57–62^. In this regard, one study showed that cortical morphology at birth reflects spatiotemporal patterns of gene expression in the fetal brain^63^, suggesting that the shape developments observed here postpartum are a direct extension of intra-uterine genetic regulation. Similarly, a recent study found that deviations from normative brain age in adulthood were best explained by congenital factors such as polygenetic risk, suggesting that early-life genetic factors exert a lifelong influence on brain structure^64^.

Finally, the strong link between genetic information and brain shape enabled us to predict which infants are twin siblings from their MRI data, identifying the brain of one twin from the brain of the other twin. Here again, FD showed the highest predictive power, outperforming not only volume as a measure of brain size but also all other morphological measures, including cortical thickness, curvature, gyrification, sulcation, surface area and the T1w/T2w ratio. Overall, these findings suggest that brain shape similarity is a direct expression of genetic similarity and that the variability of individual brain shapes represents a genetically modulated and heritable phenotype in humans.

In sum, our study identifies the early-life formation of brain shape as a fundamental maturational process in human newborns, with several immediate implications for understanding normative brain development, the study of neurodevelopmental disorders and the relationship between individual brain morphology and genetics.

## Methods

### Data and image processing

Neonatal data were obtained from the third release of the dHCP (https://www.developingconnectome.org/), including cross-sectional data for *n* = 782 infants (360 females, 422 males). The dHCP protocol was approved by the United Kingdom Health Research Authority (Research Ethics Committee reference number 14/LO/1169), and written informed consent was obtained from the legal guardian/next of kin^11^. MRI of virtually all newborns was acquired during natural sleep^11^. Mean birth age was 37.89 ± 4.17 post-menstrual weeks (range, 23.0–43.57), and age at first scan was 39.81 ± 3.55 weeks (range, 26.71–45.14). Of these infants, 682 were born from singleton pregnancies, and 100 were born from multi-fetal pregnancies. Follow-up MRI scans for longitudinal analyses were available for *n* = 100 infants. Note that, compared to adult brains, tissue contrasts in neonatal brains are inverted due to immature myelination^3,65^, such that T2-weighted images provide better quality and were hence used for image processing in the dHCP^15^. To control for potential confounds in these data, the dHCP developed a series of advanced acquisition protocols and correction schemes for neonatal MRI^15,66–68^. Specifically, motion correction and super-resolution reconstruction were achieved by combining techniques from Cordero-Grande et al.^66^ and Kuklisova-Murgasova et al.^68^, which rest on rigid-body motion estimation and motion-compensated reconstruction, resulting in isotropic volumes of 0.5-mm^3^ spatial resolution^15^. These images were subsequently passed to the neonatal processing pipeline, including correction for intensity inhomogeneity, brain extraction, tissue segmentation and surface modeling^15^. Therein, the segmentations of individual images were based on the DRAW-EM algorithm (Developing brain Region Annotation With Expectation-Maximization)^13,15^, where the assignment of individual voxels to regions of interest (ROIs) rests on the ALBERT atlases for neonatal brain anatomy (‘ALBERT: a label-based encephalic ROI template’)^12^ as modified by Makropoulos et al.^13^. This atlas contains 87 regions, including 16 cortical GM and WM regions for each hemisphere, nine bilateral subcortical regions, the brainstem and corpus callosum as unpaired regions as well as unlabeled tissue, background and cerebrospinal fluid. Here, we combined some smaller and contiguous regions to harmonize spatial granularity across the brain. Specifically, we combined the medial and lateral part of the anterior temporal lobe, the anterior and posterior segments of the gyri parahippocampalis et ambiens, the anterior and posterior lateral occipitotemporal gyrus as well as high-intensity and low-intensity voxels of the thalamus, yielding a total of 70 ROIs assigned in each MRI.

Using these data, the main focus of our study was to compare FD as a measure of brain shape and volume as a measure of brain size in their ability to capture early-life brain development. However, both of these measures are derived from a voxel-wise three-dimensional representation of the brain, raising the additional question of how FD compares to morphological measures of the cerebral cortex that are derived from surface modeling^14,15,32^. Therefore, we furthermore assessed the utility of our framework against a set of six common surface-derived measures, including cortical thickness, curvature, sulcation, the T1w/T2w ratio as well as surface area and gyrification. Note that, whereas the former four measures were directly available with the dHCP release (https://biomedia.github.io/dHCP-release-notes/structure.html#structural-pipeline), surface area and gyrification index were computed with the additional package for the dHCP structural pipeline, given here: https://github.com/amakropoulos/structural-pipeline-measures/tree/master. Complete morphological data were available for *n* = 609 infants and underlie the analyses in Figs. 3 and 8. Note that the same region labels as above were projected onto the cortical surface, such that surface measures were averaged within a parcel to obtain ROI-wise estimates. Therein, we averaged over the absolute values of sulcation and curvature, as these measures include positive and negative entries that equally carry important morphological information (for example, convexity and concavity). Finally, note that the dHCP provides age-specific normative templates by week of post-menstrual age to account for the rapid development of neonatal brains. These age-specific templates are openly available from https://brain-development.org (refs. ^14,32,69^), and the surface renderings of left cortical GM correspond to the week-wise averages displayed in Fig. 1a. For all visualizations of statistical tests, results were mapped onto the 40-week template.

### A brief note on fractal geometry

Under the traditional framework of Euclidean geometry, a straight line is attributed with a dimension of 1, a plane has a dimension of 2 and a cube is characterized by a dimension of 3. Although still broadly taught as the standard geometry today, it has long been realized that natural objects of the physical world do not adhere well to these idealized Euclidean figures. The latter was famously illustrated by Benoit B. Mandelbrot—widely regarded as the founding figure of fractal geometry—in a seminal 1967 article on the coastline paradox^20^. This paradox refers to the phenomenon that many real-world curves such as coastlines do not possess well-defined length although they represent finite physical objects. In effect, the length of the object depends on the spatial scale at which it is measured (but does not converge at increasingly smaller scales), leading to curious observations such as Portugal and Spain independently reporting the length of their shared border with a difference of several hundred kilometers^70^. At the core of this paradox lies the fact that the scaling properties of the natural object do not coincide with those expected from Euclidean geometry—or, more formally, that an object’s Hausdorff−Besicovitch dimension can exceed its topological dimension^19^. To illustrate this, consider the simple scaling law

1 $$\begin{array}{l}N(x)={x}^{-D}\end{array}$$

where *x* represents a scaling factor; *N(x)* represents the number of scaled measurement units needed to recover the original object; and *D* represents the dimension estimate. This relationship can be rewritten as

2 $$\begin{array}{l}D={-\log }_{x}N(x)=-\frac{\log N(x)}{\log x}\end{array}$$

As an example, consider a straight line that is scaled by $x=\frac{1}{2}$. We now count the number of scaled measurement units (Mandelbrot calls these ‘yardsticks’) needed to recover the original object and obtain $N(x)=2$. With the above equation, we see $D=-\frac{\log 2}{\log 1/2}=1$, as would be expected from the Euclidean notion of a line. Similarly, consider the case of a square that is covered with scaled squares of side length *x*. Here we obtain $N(x)=4$ scaled units needed to retrieve the original square. Thus, $D=-\frac{\log 4}{\log 1/2}=2$, and the case of a cube follows in analogy to yield $D=-\frac{\log 8}{\log 1/2}=3$.

Importantly, however, many objects—both in pure mathematics and the real world—do not follow this behavior. One famous example is the so-called Koch curve^71^, which possesses infinite length and scales according to $D=-\frac{\log 4}{\log 1/3}\approx 1.26$. This object is thus described by a non-integer dimension *D*, for which Mandelbrot coined the term ‘fractal dimension’ from the Latin ‘fractus’: broken, fragmented or irregular^19^. Such curves can be said to possess scaling properties that lie in between those of a line and those of a plane and are an expression of the object’s higher structural complexity compared to the Euclidean line. Notably, the term ‘complexity’ carries different connotations depending on the field of study, which can lead to misunderstandings or interpretational issues. With regard to fractal analysis, we use the term in a purely technical way: structural complexity quantifies the non-Euclidean scaling properties of the object under study. However, we realize that a more intuitive interpretation may be helpful, and, within the present context, the FD estimate can be roughly interpreted as an index of how ‘space-filling’ an object is with regard to the embedding space (here, the three-dimensional matrix representing the brain MRI). For an illustration of this geometric intuition, see the morphological simulation study below and in Extended Data Fig. 6.

In sum, the idealized dimensions of Euclid can be viewed as special cases of a more general geometry that allows for non-integer dimensions and lends itself to the analysis of naturally occurring forms in the biophysical world.

### Estimating FD from structural MRI

Although *D* can be computed in an exact fashion for objects like the Koch curve, it must be estimated empirically for real-world data (we here use the term ‘dimensionality’ instead of ‘dimension’ to reflect this distinction). To this end, the most common method is the box-counting algorithm^16,21^, in which boxes of side length *ε* are imposed on the object of interest, and *N(ε)* represents the minimum number of boxes needed to cover the object comprehensively. The FD estimate is then given by the box-counting dimension *D*_BC_ as

3 $$\begin{array}{l}{D}_{\mathrm{BC}}=\mathop{\mathrm{lim}}\limits_{\varepsilon \to 0}\frac{\log {N}({\varepsilon })}{\log (1/\varepsilon )}\end{array}$$

However, whereas theoretical fractal sets can be downscaled infinitely, the zero limit typically does not apply to empirical data (and in neuroimaging, the smallest observable scale usually corresponds to the voxel resolution^16^). Therefore, the FD estimate is in practice computed over a finite set of physical scales and is given by the slope of the regression line of box count versus box size in log−log space^16,17,21^.

Here we use a modification of this classical three-dimensional box-counting method, in which each box is replaced with a cube of a given size through iterative convolution with a set of spatial kernels, amounting to a ‘dilation’ procedure that is mathematically equivalent to applying box counting with a sliding grid^17,18^. Previous validation studies showed that this dilation algorithm represents a more robust version of classical box counting in that it (1) fares better in benchmarking studies of simulated objects^17^, (2) is less sensitive to object translation and rotation^17^ and (3) yields better test−retest reliability than classical box counting^18^. Computationally, this dilation procedure was implemented with the calcFD toolbox^17,23^ for MATLAB (MathWorks, Inc.), openly available from https://github.com/cMadan/calcFD.

To estimate the FD of brain structures from MRI data, voxels belonging to the ROI are first indexed through a segmentation procedure (here we use the abovementioned dHCP segmentation), yielding a binary three-dimensional mask that is subsequently passed to the dilation algorithm. Therein, the physical scales over which the FD estimate is computed correspond to a range of voxel sizes^16^, typically expressed as 2^*k*^ with $k\in {{\mathbb{N}}}_{0}$. Here, we follow previous applications of the toolbox in applying the range of *k* = 0, 1, …, 4 for estimation with the dilation algorithm^17,18,23,25^. For each of these spatial scales, the three-dimensional convolution of the index mask is calculated, resulting in a volumetric count in relation to the spatial scale. Extended Data Fig. 1 illustrates how this dilation procedure is used to compute the FD estimate from the scaling properties of a voxel-indexed segmentation mask. In this example, FD is estimated for global segmentations of cortical GM and WM in two recordings of a representative neonate. Note that we use global tissue segmentations for the analyses in Extended Data Figs. 5 and 7–10, whereas the analyses in the main text were carried out in the parcellated brain data as described above, yielding one FD estimate per region and thus a 1 × 70 vector for every scan. This region-wise FD estimation follows the same procedure and is illustrated for the left parietal cortex of the same exemplary infant in Fig. 1b.

### Inferential statistics and modeling

All directional tests were two-tailed. Simple two-group comparisons were tested with *t*-tests or rank-sum tests, depending on the distribution of the variables, and in analogy for correlational analyses with either product−moment or Spearman’s rank correlation. Two-sample tests were unpaired, unless stated otherwise (for example, the longitudinal analyses in Fig. 4a in which each newborn had a baseline and a follow-up scan, representing paired samples). Effect sizes for parametric group tests were computed as Cohen’s *d*. Parametric correlation strengths were Fisher *r*-to-*z*-transformed to harmonize scales for visualization (for example, Fig. 2a). Multiple-group omnibus tests were implemented with Kruskal−Wallis tests, followed up by pairwise Dunn’s tests. Formal significance was considered at an *α* level of 0.05, and *P* values of multiple pairwise tests were corrected after Benjamini−Hochberg^72^ to control the FDR. For the statistical comparison of correlation coefficients in dependent groups (Fig. 2), the null hypothesis posits that two variables (for example, FD and volume) are equally correlated with a third variable (for example, age), all obtained from the same individuals, which is testable through Williams’ *t*-statistic^73,74^. For model comparisons across different brain measures (Figs. 3a,b and 8b), we implemented a permutation approach on the effect estimate given *n* empirical observations of *m* variables. The null hypothesis under this regime posits that there is no difference in the observed effect between two brain measures X and Y and that, consequently, the effect attributed to observations of X can be equally attributed to the corresponding observations of Y. To test this hypothesis, we first standardize observations in X and Y to the same scale, using *z*-scores for parametric models and ranks for non-parametric models. Subsequently, we choose $\frac{n}{2}$ observations of X at random and replace these data points with the corresponding observations in Y, yielding a new variable $\bar{X}$. We then estimate the statistic of interest on $\bar{X}$ and repeat this process many times to obtain a null distribution of the statistic, which approximates the assumption that it does not matter if observations belong to X or Y. The empirical estimate is then compared to this null distribution, where the ensuing *P* value is given by the proportion of permuted estimates equal to or greater than the empirical estimate over the number of permutation iterations (here, *n* = 10,000). This procedure equally applies to *m* = 1 (that is, **X** and **Y** are vectors) and *m* > 1 (that is, *X* and *Y* are matrices), where random replacements are applied per column in the latter case.

Moreover, the hierarchical clustering grouping in Fig. 3c was tested with the sigclust package for R^75^. Furthermore, for the FD covariance network in Extended Data Fig. 2, the pairwise region-to-region correlation matrix of FD values was constructed from the cross-sectional scans in Fig. 2, and this matrix was thresholded to the top and bottom first percentile to obtain the strongest positive and inverse covariance across brain regions. Additionally, the hierarchical regression in Extended Data Fig. 4 compared a compact model in which the FD of a brain region was explained with infant age alone (FD~age) to two augmented models that incorporated sex (FD~age+sex) and pregnancy status (FD~age+pregnancy), respectively. To estimate in which brain regions these factors significantly explained additional variance beyond age differences, compact and augmented models were compared with *F*-tests for nested models, using the lmSupport package for R (https://rdrr.io/cran/lmSupport).

### Replication analyses

The replication analyses in Extended Data Figs. 5, 7 and 9 were carried out in an independent dataset of human newborns from the University of California, Irvine (UCI) (ethics approval no. 2009-7251)^33,34^. The UCI data comprised *n* = 99 newborns born to healthy pregnancies with no known major complications. The sex distribution in the UCI sample was largely balanced (*n* = 48 females (48.5%); *n* = 51 males (51.5%)) and did not differ significantly from the dHCP sample (*χ*^2^ = 0.21, *P*= 0.65). However, infants in the UCI cohort were significantly older on average (42.87 ± 2.01 post-menstrual weeks (range, 39.57–48.57), *z* = 8.47, *P*< 0.001; Extended Data Fig. 5a). Besides the age range and geographic locations of the study sites (dHCP: United Kingdom; UCI: United States), noteworthy differences include the scanner type (dHCP: 3T Philips Achieva; UCI: 3T Siemens TIM Trio), acquisition parameters and processing software^15,33,34^, the post-reconstruction spatial resolution (dHCP: 0.5 mm^3^; UCI: 1 mm^3^) and the parcellation approaches^14,15,33^.

Replication analyses thus focused on global tissue segmentations of cortical GM and WM to ensure comparability and address the potential impact of arbitrary tissue cuts due to a particular parcellation template. Accordingly, we estimated the FD of these global segmentations and computed the product−moment correlation to age at scan for each tissue class and dataset separately (Extended Data Fig. 5b). The direction of age−FD effects was then assessed by the sign of the estimate, testing if it significantly differed from zero in both datasets. Furthermore, the magnitude of age−FD associations in the two cohorts was statistically compared through a *z*-statistic obtained from correlation coefficients of independent groups^74^ and computed with the cocor package for R (http://comparingcorrelations.org/).

### Morphological simulation study

Moreover, we implemented a morphological simulation study to illustrate the geometric interpretation of FD and help explain the tissue-specific direction of the empirical age−FD effects (Extended Data Fig. 6a). As detailed above, Euclidean geometry attributes a plane with a dimension of 2, whereas a cube is characterized by a dimension of 3. These idealized structures thus represent special cases of equation (2), where the exponent of the spatial scaling law resolves to a positive integer. Therefore, the rationale behind the simulation study was to illustrate how FD maps the spectrum between these Euclidean special cases by gradually transforming one into the other. Specifically, the simulation starts from a plane (theoretical FD = 2) and slowly ‘grows’ into a cube (theoretical FD = 3) through a series of random additions. To this end, we first construct a binary matrix of 0s (100 × 100 × 100 voxels) into which we insert a plane of 1s (100 × 100) as the initial object. We then index all voxels that are on the surface of the object and randomly choose one surface voxel as the center of a 5 × 5 × 5 cube that we set to 1. We then repeat this process over many iterations, gradually filling the matrix with 1s until we arrive at a volume of all 1s (that is, the cube). Here, we set the number of iterations to 30,000 and repeated the simulation 100 times. With this approach, all simulation runs started from the same plane and arrived at the same cube, but the objects in between varied. In each iteration, we computed the FD of the simulated object exactly as outlined for the empirical data. Furthermore, we hypothesized that the FD of the simulated objects would show a principled link to their SVRs, computed as the sum of surface voxels over the sum of all voxels of the object. The idea behind this approach is that FD can be intuitively interpreted as an index of how ‘space-filling’ the object is relative to the embedding matrix. For illustration, the initial plane is the least space-filling object in the simulation, whereas its SVR is maximal because all voxels are surface voxels (that is, SVR = 1). By contrast, the final cube completely fills the embedding matrix, whereas its SVR is minimal because most voxels are inside the cube. Given the matrix dimensions, the number of unique surface voxels of the full cube amounts to 58,800, such that the expected SVR of the final cube yields 0.0588. All simulation runs returned the theoretically expected FD and SVR values, both for the plane and for the cube (Extended Data Fig. 6b). Finally, it is worth mentioning that Extended Data Fig. 6 shows the results of transforming the plane into the cube. As a further control analysis, we also implemented the opposite transformation (that is, transforming the cube into the plane), with virtually identical results (FD versus SVR for plane to cube: *r* = −0.994, *P* < 0.001; for cube to plane: *r* = −0.985, *P* < 0.001).

### Further validation analyses

Given the results of the simulation study, we furthermore tested if the FD−SVR relationship would also be observed in the empirical brain data (Extended Data Fig. 7). Therefore, we ran a series of correlation tests between FD and SVR values in the dHCP regional parcellations (Extended Data Fig. 7a) as well as global tissue segmentations for the dHCP and UCI data (Extended Data Fig. 7b, upper and lower row, respectively).

Moreover, we implemented an additional control analysis to test if the inverse age−FD in WM could be flipped by artificially manipulating the images to be more similar to the GM segmentations (Extended Data Fig. 9). To this end, we hollowed out the original segmentations and computed FD values from these hollowed images (Extended Data Fig. 9a). Notably, FD values from hollowed data were universally lower than those computed from the original segmentations, as expected from previous validation studies^17,23^. Moreover, given the geometric interpretation of the age−FD effects in GM and WM (Extended Data Figs. 6 and 8), we hypothesized that the impact of the hollowing procedure would depend on the age of the infants. Therefore, we computed the numerical effect of the procedure as ${\Delta {\rm{FD}}={\rm{FD}}}_{{\rm{original}}}-{{\rm{FD}}}_{{\rm{hollow}}}$ and correlated this difference with age at scan (${r}_{\Delta \mathrm{FD}}$ in Extended Data Fig. 9c,d).

Additionally, we conducted the analyses in Extended Data Fig. 10 to validate WM volume and FD against a biophysical proxy of WM microstructure. To this end, we estimated the ratio of T1w/T2w data in all voxels of the WM border^15,76^. This estimation was based on the bias-corrected T1-weighted images, the bias-corrected T2-weighted images and the corresponding tissue segmentation (data available for *n* = 631 infants), which were all provided in T2-weighted space by the dHCP (Extended Data Fig. 10a). This approach allowed us to sample voxels inside the WM mask (amounting to 1 mm, panel **a**, right) and to extract the T1w/T2w intensities in the corresponding voxel locations. The respective ratio of these intensities then yielded a voxel-wise T1w/T2w map (Extended Data Fig. 10b), and the median across all indexed voxels was computed as a summary proxy of WM microstructure for every infant (Extended Data Fig. 10c). Notably, the quantitative range of T1w/T2w ratios observed here is highly consistent with a recent study that used a similar approach to estimate the microstructural developments of WM bundles in the dHCP cohort^77^. The relationship between the T1w/T2w proxy and WM volume and WM−FD, respectively, was then assessed by (1) statistically comparing the absolute effect sizes obtained from dependent groups (as described above; Extended Data Fig. 10d, left) and by (2) computing partial correlations across all three variables (Extended Data Fig. 10d, right) using the ppcor package in R^78^.

### Predicting infant age

The age prediction pipeline in Fig. 5 rests on the openly available PRISM toolbox (https://github.com/cMadan/prism) for MATLAB, which was developed for age prediction from brain features and includes a combination of least squares splines, dimensionality reduction and relevance vector regression^25,35^. Here, the smoothing parameter for spline regression was set to zero, enforcing near least squares cubic spline to counteract overfitting; all other parameters were left to default, including the application of principal component analysis and relevance vector regression within a sparse Bayesian framework^79^. The predictor matrix was of the form (observations × brain features) and contained either FD values, volumes or both. All predictors were standardized. To evaluate prediction performance, we applied a 10-fold cross-validation scheme, such that the model was trained on 90% of the data and predicted age at scan in the remaining 10% of the data in each iteration. Note that, here, we limited the dataset to the 782 unique baseline scans (that is, excluding the follow-up sessions) to ensure that every infant contributed exactly one scan to the data. Prediction quality for each iteration was then assessed as the MAE (|predicted age − true age|) and the variance explained in the test set (*R*^2^ = 1 − residual sum of squares / total sum of squares), as shown in Fig. 5b. For the random repetitions of the cross-validation procedure (Fig. 5c), we computed 500 unique permutations of the data that were subsequently split into 10 folds, resulting in 5,000 predictions on unique test sets. Performance differences between FD and volume were tested with signed-rank tests. Finally, to assess the impact of different model types, we applied the same prediction pipeline using simple multiple linear regression and support vector regression with a linear kernel with MATLAB-inbuilt functions (fitlm and fitrsvm), as shown in Supplementary Fig. 1.

### Departure from normative reference

For the analyses in Fig. 6, we estimated reference values of brain shape and size in infants of full-term maturity. This approach is conceptually related to the hub disruption index^80^ in functional neuroimaging, in that data points from single individuals are compared to normative data points obtained from a reference population. Here, the reference population consisted of those infants who were both born and scanned within the full-term window (term−term), where the latter was defined based on the ACOG definitions (39 0/7 weeks to 40 6/7 weeks). This criterion was fulfilled by *n* = 116 newborns in the dataset. For each brain region, the full-term reference value was then computed as the average over those 116 infants, once for FD values (shape reference; Fig. 6a) and once for volumes (size reference). This approach subsequently allowed for a comparison between the reference values across all brain regions and the corresponding values computed from individual scans, as shown in the scatter plots of Fig. 6b. To estimate how much these individual scans deviated from the full-term reference, we computed a departure index defined as *d* = 1 − *ϱ* (that is, the non-parametric spatial correlation distance between the individual scan and the normative reference). Therein, Spearman’s rank correlation was chosen because (1) we aimed to obtain an estimate of the relative spatial organization across the whole brain and because (2) the speed of development varied over the different tissue classes (Fig. 4d), such that the deviations from reference were not uniform but showed clustering effects (for example, deviations cluster below the identity line in Fig. 6b). For each scan, we thus obtain one index of departure from full-term shape reference (FD) and another for the departure from full-term size reference (volume). These indices were subsequently compared across all scans (Fig. 6c) and among infants who were born preterm and scanned preterm (preterm−preterm) and those who were born preterm but scanned later at term-equivalent age (preterm−term) (Fig. 6d). Finally, note that infants who met the full-term criterion are expected to follow the reference closely because they formed part of the group on which this reference was defined, thus providing an estimate of variability within the full-term group itself. This close adherence to reference was indeed observed for both FD and volume in full-term infants (Fig. 6b–d).

The above framework thus implemented a normative account of morphological developments on the level of single infants, reflecting individual patterns of whole-brain deviations that are not easily captured by group-level analyses. However, we complemented this framework by an exploratory approach on the FD features, which (1) explicitly captures the region-by-region variability within the term−term reference group and (2) quantifies the group-level deviation of individual brain features for preterm−preterm and preterm−term infants. These analyses are summarized in Supplementary Fig. 3. Therein, for any given brain region, we extract the distribution of FD values for the term−term group, which allows for an estimation of the variability within the norm as the standard deviation (s.d.) over the respective vector (Supplementary Fig. 3a, right). Furthermore, this approach allowed us to compute *z*-scores with respect to the reference as

4 $$\begin{array}{l}{z}_{i,\,j}=\frac{{x}_{i,\,j}-{\mu }_{j,\mathrm{term}-\mathrm{term}}}{{\sigma }_{j,\mathrm{term}-\mathrm{term}}}\end{array}$$

where *x*_*i,j*_ represents the FD value of the *i*-th infant in the respective target group (preterm−preterm or preterm−term) and in the *j*-th brain region; ${\mu }_{j,\mathrm{term}-\mathrm{term}}$ represents the mean FD value in that brain region over the term−term reference group; and ${\sigma }_{j,\mathrm{term}-\mathrm{term}}$ represents the corresponding s.d. in the reference group. In Supplementary Fig. 3a (left), the computation of these norm-referenced *z*-scores is illustrated for the example region of left frontal GM. Here, we display one infant of the preterm−preterm group whose *z*-score corresponds to the left tail of the term−term distribution (blue) and one infant of the preterm−term group whose *z*-score corresponds to the right tail of the reference distribution (red). This approach subsequently allowed us to collect the *z*-scores for all infants of the preterm−preterm and preterm−term group and test if the distribution of these *z*-scores was significantly different from zero (one sample *t*-test). Panels **b** and **c** of Supplementary Fig. 3 show the results of these analyses across all brain regions for the preterm−preterm and preterm−term groups, respectively.

### Comparing individual infant brains

To move beyond group-level inferences, we conducted comprehensive pairwise ‘brain-to-brain’ comparisons of individual neonates (Figs. 7 and 8). For any two given infants, we thus quantified the overall ‘shape difference’ of their brains by taking the vectors of their regional FD values and computing the dissimilarity between these two vectors. To this end, we here apply the L1 norm (‘Manhattan distance’), as this measure weights all vector components equally and is less sensitive to single-dimension deviations compared to the Euclidean distance, because the individual terms are left unsquared. For every brain-to-brain comparison, this approach yields a scalar measure of overall dissimilarity (Fig. 7a), such that higher values indicate more pronounced shape differences and lower values indicate that the compared brains are more similar in shape. Moreover, the identical approach was applied to regional volumes to compute the overall dissimilarity in size between any two brains (Supplementary Fig. 5) and likewise for the systematic comparisons to surface-derived measures in Fig. 8.

### Genetic similarity

These brain-to-brain comparisons subsequently allowed us to relate the shape similarity of any two brains to the genetic similarity of the compared infants. The latter was formalized in three different sets of comparisons: (1) infants of the same sex versus infants of different sexes (as assigned at birth; Supplementary Fig. 4); (2) twin siblings versus unrelated infants (Figs. 7c,d and 8c); and (3) identical twins versus fraternal twins (Figs. 7e,f and 8c). Overall, there were 42 twin pairs in the dHCP dataset. For the age-matched analyses in Fig. 7, however, a total of seven twin pairs had to be discarded—one because no unrelated infants of the same age were available and six because the two twin siblings themselves were scanned more than 1 day apart—leaving *n* = 35 twin pairs. Moreover, the genetic similarity among those twin pairs was further assessed by stratifying them into identical twins (that is, monozygotic siblings) and fraternal twins (that is, dizygotic siblings). This information on twin status was provided by the dHCP consortium (Harriet Cullen, King’s College London) and was derived from single-nucleotide polymorphism array genotype data, which were used to confirm whether the twins were monozygotic, sharing 100% of their genetic variation (PI_HAT = 1), or dizygotic, sharing approximately 50% of their genetic variation (PI_HAT ≈ 0.5)^81^. These data on twin sibling status were available for *n* = 33 twin pairs.

### Twin predictions

Apart from the inferential analyses of sex and twin status, we furthermore predicted twin siblings out of the set of age-matched unrelated infants in a supervised approach (Figs. 7f and 8c). To this end, we iterated over all individual twins-to-unrelated comparisons and predicted the lowest-ranking dissimilarity score (that is, the most similar brain in shape) to belong to the twin of the target infant, as detailed in Fig. 7c. Note that, although the set of unrelated matches was the same for a given twin pair, the dissimilarity scores between twin A and the unrelated infants and twin B and the unrelated infants naturally differed, as all these comparisons reflect individual pairwise brain-to-brain measures. In consequence, every twin pair resulted in two predictions—once identifying twin A from twin B and once identifying twin B from twin A—yielding 70 twin predictions in total. Furthermore, note that the number *n* of unrelated matches varied across the individual twin pairs, such that the chance level of individual twin predictions varied in parallel as 1 / *n*. For illustration, the example of Fig. 7c features 13 age-matched infants (one of whom is the twin to be identified), resulting in a chance level of $1/13\approx 7.7 \%$. As such, chance levels for individual predictions were higher if fewer unrelated matches were available in the dataset (maximum 50% if only one unrelated match was present). To account for this heterogeneity, we implemented a permutation approach, in which the rank structure within individual predictions was randomly shuffled 5,000 times and the proportion of chance identifications was recorded over all individual predictions. In consequence, we obtain a null distribution of correct twin identifications that happen by chance, which yields the *P* value of the empirically observed identification accuracy as the proportion of permuted accuracies that surpass the empirical value. The inset of Fig. 7f shows this null distribution, which yielded a mean accuracy of $11.4\pm 3.7 \%$ of correct twin identifications that are expected to happen by chance.

Finally, the identical approach was applied to twin prediction from brain volumes (Supplementary Fig. 6) as well as surface-derived measures (Fig. 8).

### Reporting summary

Further information on research design is available in the Nature Portfolio Reporting Summary linked to this article.

## Online content

Any methods, additional references, Nature Portfolio reporting summaries, source data, extended data, supplementary information, acknowledgements, peer review information; details of author contributions and competing interests; and statements of data and code availability are available at 10.1038/s41593-025-02107-w.

## Supplementary information

Supplementary Information Supplementary Figs. 1−6 and supplementary text

Reporting Summary

## Source data

Main and Extended Data Figures Statistical Source Data.

## Data Availability

Data used in the preparation of this paper were obtained from the National Institute of Mental Health (NIMH) Data Archive (NDA). The NDA is a collaborative informatics system created by the National Institutes of Health (NIH) to provide a national resource to support and accelerate research in mental health. Dataset identifiers are as follows: Collection ID 3955 (dHCP) and Collection ID 1890 (UCI). This paper reflects the views of the authors and may not reflect the opinions or views of the NIH or of those submitting original data to the NDA. Please note that the direct sharing of raw data or derivatives by the authors is not permitted as per NDA policy. However, researchers can independently obtain data access at the NDA using the above Collection IDs or the corresponding study DOIs^82,83^. For further information, please see NDA study 3107 (10.15154/jdep-kf48). Source data are provided with this paper.

## Code availability

Analysis code supporting the findings of this study is available from the corresponding authors and the Open Science Framework (https://osf.io/6jck4/).
