# Supplementary material for: Fractal analysis of brain shape formation predicts age and genetic similarity in human newborns
Source: Nat Neurosci. 2025 Dec 29;29(1):171–85. doi: 10.1038/s41593-025-02107-w (PMC12779576; doi:10.1038/s41593-025-02107-w)
Supplement: Supplementary file 1 — Supplementary Figs. 1−6 and supplementary text [file 41593_2025_2107_MOESM1_ESM.pdf]

nature neuroscience

Supplementary information https://doi.org/10.1038/s41593-025-02107-w

Fractal analysis of brain shape formation predicts age and genetic similarity in human newborns

In the format provided by the authors and unedited

Supplementary Figures 1-6

Supplementary Text

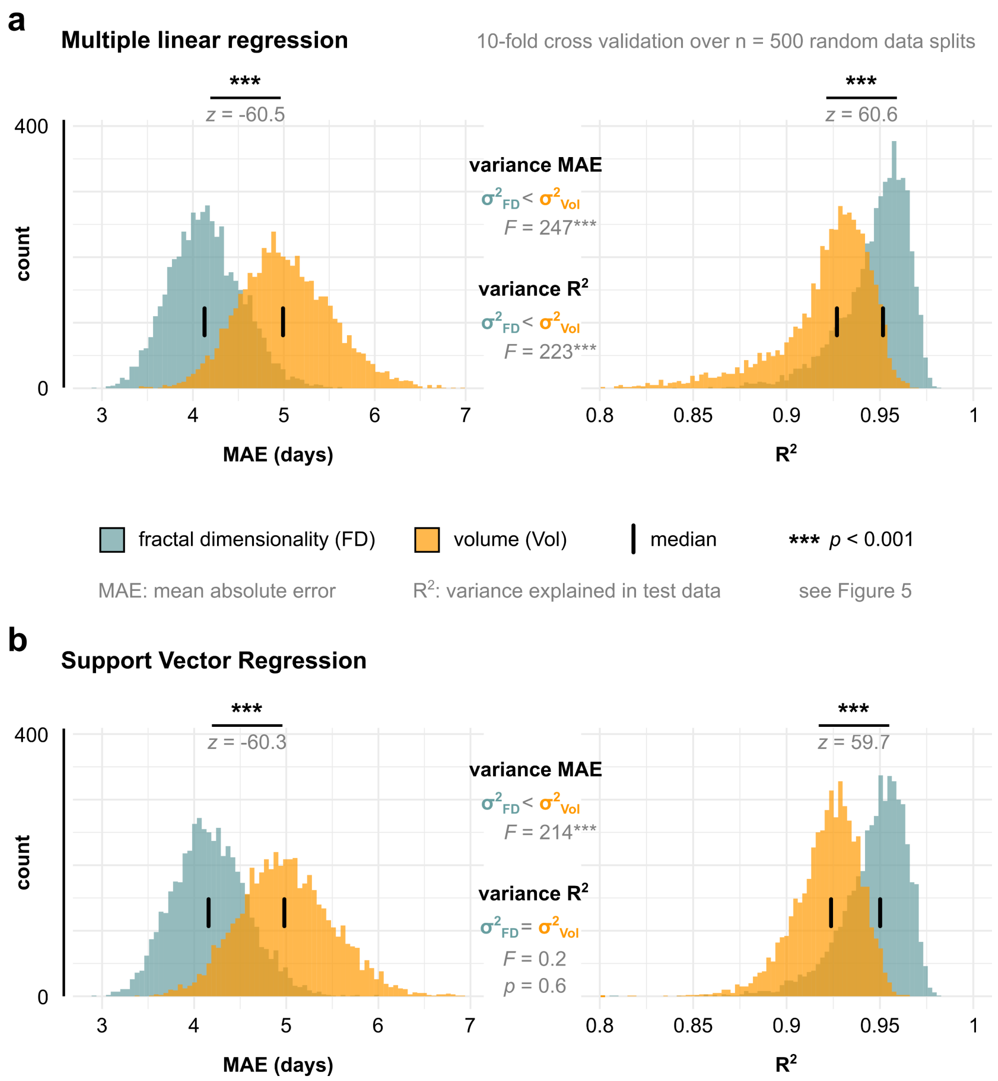

Supplementary Fig. 1 | The superior performance of age prediction from fractal dimensionality (FD) over age prediction from volume is equivalently observed in two alternative control models. **a**, Results of running the age prediction pipeline outlined in main Figure 5a with simple multiple linear regression. The distributions of performance metrics (MAE: mean absolute prediction error in days; R^2: variance explained in test data) are obtained from 500 random repetitions of the 10-fold cross validation procedure and compared with signed-rank tests ($p \approx 0$ within machine precision for both MAE and R^2) and Levene's test (MAE: $p=5.0*10^{-55}$; R^2: $p=7.1*10^{-50}$), as in Figure 5c. **b**, Running the age prediction pipeline with Support Vector Regression (linear kernel), assessed with signed-rank tests ($p \approx 0$ within machine precision for both MAE and R^2) and Levene's test (MAE: $p=4.7*10^{-48}$; R^2: $p=0.640$), as above. All tests two-sided. Across both these simpler modelling approaches, shape-based age prediction with fractal dimensionality (FD) consistently outperformed size-based age prediction with volume (Vol).

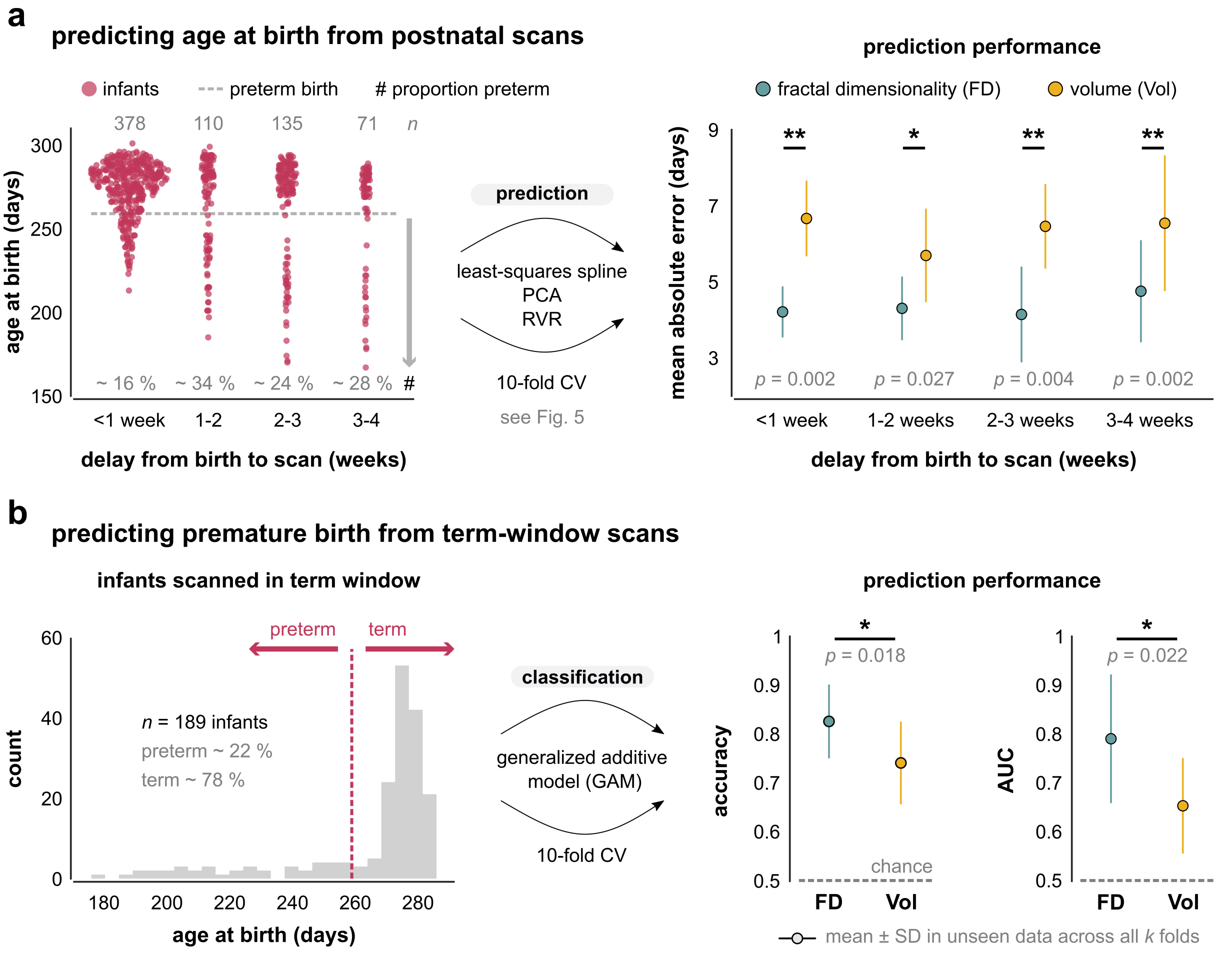

Supplementary Fig. 2 | Predicting age at birth from postnatal scans. a, Results of running the age prediction pipeline of main Figure 5a when replacing infant age at *scan* with infant age at *birth* as the target variable. Due to variable delays between birth and first scan, these analyses were stratified into scans conducted within <1, 1-2, 2-3, and 3-4 weeks post-partum, respectively (left panel). Note that (i) the <1 week group thus approximates the analyses in Figure 5, and (ii) the relative proportion of prematurely born infants is higher in the longer-delay groups, as preterm infants were less often scanned within one week after birth. Age at birth was subsequently predicted from either regional volumes (Vol) or fractal dimensionality (FD) values, using a 10-fold cross-validation scheme, as in the main text. The right panel shows prediction performance as the mean absolute prediction error in days ± standard deviation in unseen data. FD significantly outperformed Vol in all delay groups, assessed with two-sided signed-rank tests. **b**, Predicting preterm vs term birth from scans acquired in the term window. Since the distribution of birth age for these scans was heavily skewed (left panel), we implemented a supervised binary classification approach (preterm vs term birth) based on generalized additive models (GAM), employing a 10-fold cross-validation as above. The right panels show the accuracy and area under the curve (AUC) of predictions in unseen data, where FD again outperformed Vol (two-sided signed-rank tests).

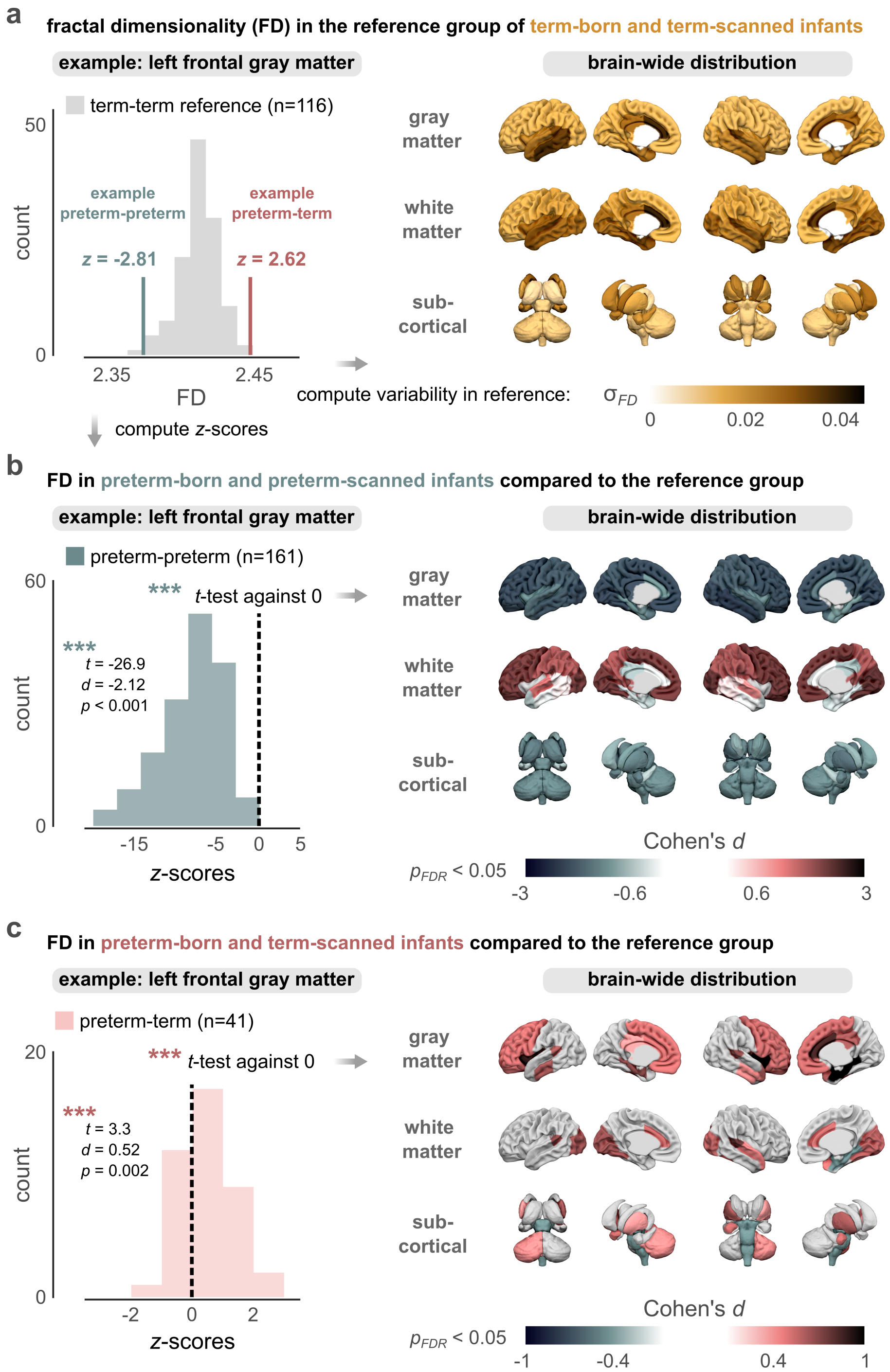

Supplementary Fig. 3 | Regional differences in normative age comparisons of fractal dimensionality (FD). a, Estimating the variability of regional FD values in the reference group of infants that were both born and scanned within the full-term window (*term-term*; see main Fig. 6). The left panel illustrates the distribution of FD values for the example region of left frontal gray matter. The variability of FD values in the term-term reference was estimated as the standard deviation σ_{FD} of this distribution for each brain region, as shown in the brain map on the right. Furthermore, the FD distribution in the reference group served the computation of infant- and region-wise *z*-scores for those infants born preterm and scanned preterm (*preterm-preterm*; example infant in blue; panel b) and those born preterm but later scanned in the full-term window (preterm-term; example infant in pink; panel c). **b,** Distribution of FD *z*-scores in the *preterm-preterm* group compared to the *term-term* reference, illustrated for the example brain region (left panel). These distributions are then tested against zero (two-sided one sample *t*-test with FDR adjustment; example region: p=2.3*10^{-61}; p_{FDR}=1.8*10^{-60}), and the ensuing effect sizes are plotted in brain space (right panel, thresholded to p_{FDR}<0.05). **c,** Distribution of *z*-scores in the *preterm-term* group. Two-sided one sample *t*-tests against zero with FDR adjustment, as above.

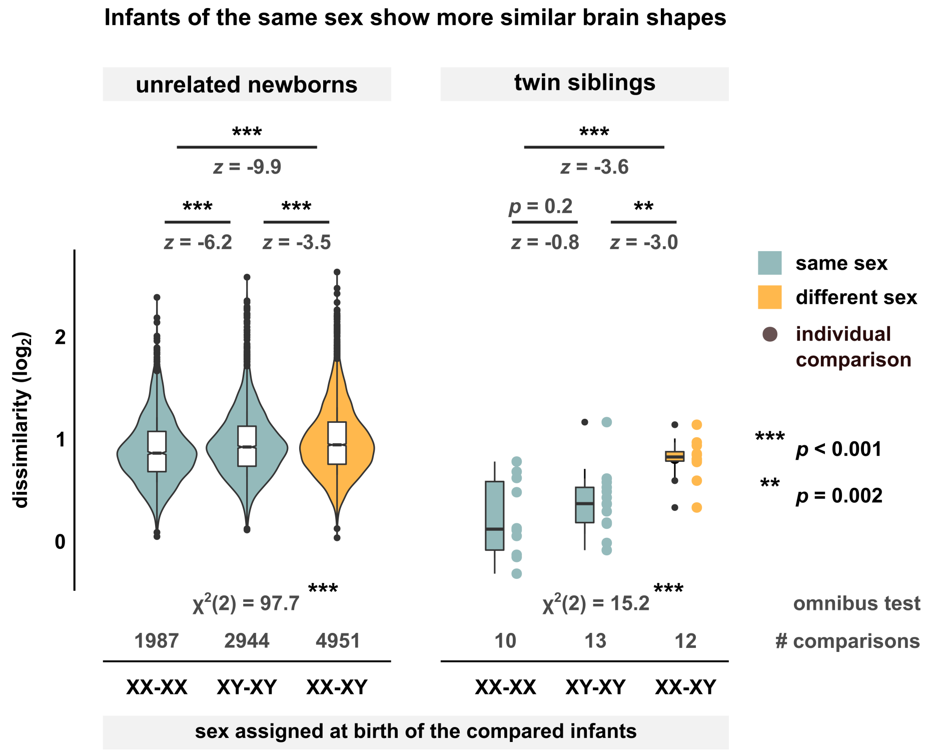

Supplementary Fig. 4 | The shape similarity of any two brains depends on the sex of the compared infants. Fractal dimensionality (FD) dissimilarity scores of all comparisons between newborns that were within 1 day of age at the time of scanning (left: unrelated infants, right: twin siblings). Dissimilarity is displayed on a binary logarithmic scale and is stratified by the sex of the compared infants (XX-XX: both infants female; XY-XY: both infants male; XX-XY: one female, one male). Omnibus tests correspond to Kruskal-Wallis tests (unrelated infants: p=6.1*10^{-22}; twins: p=4.9*10^{-4}), and pairwise comparisons to Dunn's tests with FDR adjustment (unrelated infants: female-female vs female-male: p_{FDR}=7.7*10^{-23}; male-male vs female-male: p_{FDR}=2.2*10^{-4}; male-male vs female-female: p_{FDR}=3.7*10^{-10}; Twins: female-female vs female-male: p_{FDR}=4.5*10^{-4}; male-male vs female-male: p_{FDR}=1.8*10^{-3}; male-male vs female-female: p_{FDR}=0.216). Effects remained virtually unchanged after adjustment for differences in total brain volume. Boxes in the right panel display the interquartile range (IQR; lower hinge: 25th percentile; upper hinge: 75th percentile; center line: median) and whiskers cover the furthest data points within 1.5*IQR.

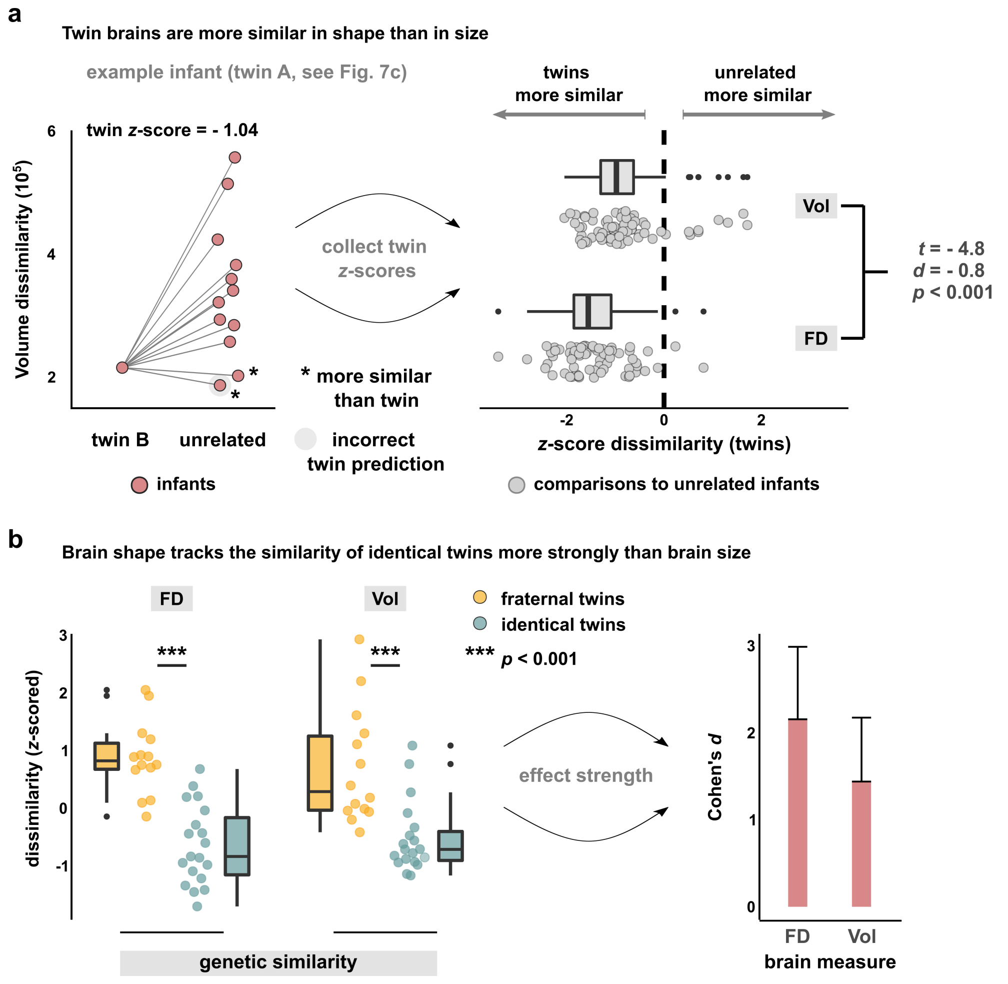

Supplementary Fig. 5 | Brain size is less sensitive to genetic similarity than brain shape. a, Dissimilarity of brain volumes (Vol). Comparisons of the exemplary infant in main Figure 7c to its twin sibling and the set of age-matched unrelated infants. Note that in contrast to the results with fractal dimensionality (FD), two unrelated newborns are more similar to the exemplary infant than its twin sibling in terms of brain size. Consequently, with volume, an unrelated infant is incorrectly predicted to be the twin of the exemplary infant, as twin prediction is based on the lowest-ranking dissimilarity score (see Supplementary Fig. 6 for a comprehensive comparison of shape-based vs. size-based twin prediction). The right panel compares the dissimilarity scores for all twin-to-unrelated comparisons between volume and FD (Welch's two-sample *t*-test, two-sided; p=4.1*10^{-6}), where the latter corresponds to the distribution of main Fig. 7d. **b**, Standardized dissimilarity scores for volume and fractal dimensionality. While both measures yield lower dissimilarity for identical twin pairs (n=19) compared to fraternal twin pairs (n=14; Welch's two-sample t-test, two-sided; FD: p=3.2*10^{-7}, Vol: p=6.4*10^{-4}), the strength of this effect is higher for FD than for volume (error bars: 95% confidence interval; center: effect size of group differences). Boxes in panels (a) and (b) display the interquartile range (IQR; lower hinge: 25th percentile; upper hinge: 75th percentile; center line: median) and whiskers cover the furthest data points within 1.5*IQR.

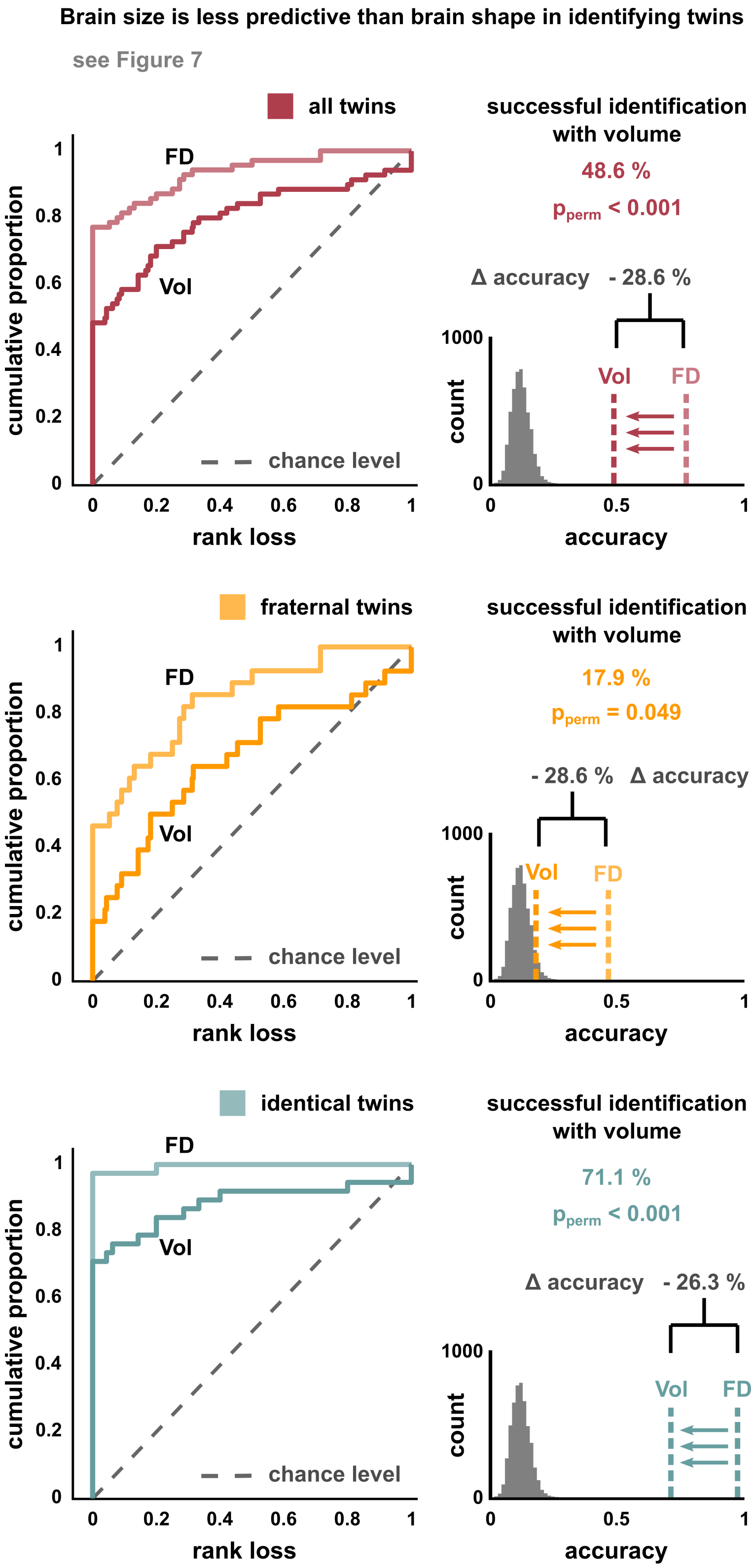

Supplementary Fig. 6 | Brain size is less predictive than brain shape in identifying twin siblings. The figure relates the predictive capacity of identifying twin siblings with volume (Vol) instead of fractal dimensionality (FD). Compared to twin prediction from brain shape (see main Fig. 7f), twin prediction from brain size showed a consistent 25-30% drop in identification accuracy for all twin predictions (top row), the subgroup of fraternal twins (middle), and the subgroup of identical twins (bottom). Gray histograms represent the null distribution of correct twin identifications that happen by chance, as in Figure 7f. *P*-values derived by permutation test, given by the number of permuted accuracies that surpass the empirically observed accuracy, divided by the number of permutations (n=5000; overall, p_{perm}=0; identical, p_{perm}=0, fraternal, p_{perm}=0.049).

Supplementary Text

Below we provide details on the validation analyses in the main text and the statistical results in Figures 3 and 8.

Explaining the tissue-specific direction of age-FD effects

The following section provides details on the validation analyses in Extended Data Figures 5-10. As detailed in the main text, the analyses in Figures 2 and 4 revealed a consistent spatial pattern of age-FD effects, which was observed both cross-sectionally and longitudinally and in which more mature brains are characterized by higher FD in GM and lower FD in WM. To explain this tissue-specific effect direction, we conducted a series of six follow-up analyses:

First, we replicated the direction of age-FD effects in an independent external validation cohort[1,2] (Extended Data Fig. 5). These data were collected at the University of California, Irvine (UCI) and comprised n=99 neonates. While the UCI cohort did not differ from the dHCP in terms of sex ratios (48.5 % female, 51.5 %; $\chi^2 = 0.21$, $p = 0.65$), the UCI infants were slightly older (42.87 ± 2.01 weeks [range: 39.57 – 48.57]; $z = 8.47$, $p<0.001$; Extended Data Fig. 5a). For comparability across datasets, we analyzed global segmentations of cortical GM and WM and uniformly observed positive associations with age for GM (dHCP: $r = 0.96$, $t = 98.0$, $p<0.001$; UCI: $r = 0.85$, $t = 15.9$, $p<0.001$) and negative associations for WM (dHCP: $r = -0.90$, $t = -58.8$, $p<0.001$; UCI: $r = -0.58$, $t = -7.0$, $p<0.001$), with effects more pronounced in the dHCP (GM: $\Delta r = 0.11$ [CI: 0.06, 0.18], $z = 6.5$, $p<0.001$; WM: $\Delta r = -0.32$ [-0.47, -0.21], $z = -7.6$, $p<0.001$). The direction of age-FD effects thus closely replicated in the validation data – independent of study site, scanner type, acquisition protocol, spatial resolution, age range, and parcellation scheme.

Second, we conducted a morphological simulation study to illustrate the geometric interpretation of FD (Extended Data Fig. 6). To this end, we focused on two idealized Euclidean objects: a plane (theoretical FD = 2) and a cube (theoretical FD = 3), computationally represented in a binary 3D matrix. Specifically, the simulation starts from the plane and gradually transforms it into the cube through random 'growth' (Extended Data Fig. 6a). This procedure thus yields an array of simulated objects whose geometric properties lie in between those of the plane and those of the cube (see Methods). Consequently, the dimension of the simulated objects is given by $2 \leq$

$FD \geq 3$, spanning a continuum between a more 'plane-like' geometry (FD towards 2) and a more 'cube-like' geometry (FD towards 3; Extended Data Fig. 6b). Additionally, the simulation showed a strong inverse relationship between the FD of the simulated objects and their surface-to-volume voxel ratios (SVR), where the latter can be viewed as a proxy for how 'space-filling' the object is with regard to the embedding matrix. For illustration, the least space in the simulation is occupied by the initial plane (FD = 2), whose SVR is maximal (because all voxels are surface voxels). In contrast, the final cube completely fills the embedding matrix (FD = 3), while the SVR is minimal (because most voxels are inside the cube). Accordingly, a lower FD can be interpreted to indicate that the object is less space-filling, while a higher FD indicates the object fills more of the embedding matrix. In this context, it is important to note that this relationship is independent of absolute size (and thus volume) – a cube of half or double the size equivalently yields FD = 3 because the underlying scaling exponent is identical (see Methods).

Third, we tested if this theoretical association between FD and SVR in simulated objects would also be present in the empirical brain data (Extended Data Fig. 7). Indeed, the FD-SVR relationship was (i) consistently observed across all regional parcels in the dHCP (Extended Data Fig. 7a), was (ii) corroborated in global tissue segmentations (Extended Data Fig. 7b, top), and (iii) replicated in the validation data (Extended Data Fig. 7b, bottom). This held true for both GM (dHCP: $r = -0.90$, $t = -56.7$, $p<0.001$; UCI: $r = -0.69$, $t = -9.4$, $p<0.001$) and WM (dHCP: $r = -0.97$, $t = -111.2$, $p<0.001$; UCI: $r = -0.83$, $t = -14.4$, $p<0.001$), where effects were again stronger in the dHCP cohort (GM: $\Delta r = -0.21$ [-0.32, -0.11], $z = -5.6$, $p<0.001$; WM: $\Delta r = -0.14$ [-0.22, -0.09], $z = -8.5$, $p<0.001$).

Fourth, we related the geometric interpretation of the simulation study to the empirical FD and SVR values (Extended Data Fig. 8). Therein, we observed that the FD values of GM and WM numerically converge towards term maturity (Extended Data Fig. 8a), with a GM-WM equilibrium point around 40-42 weeks. Accordingly, comparing preterm-window and term-window scans showed that more mature brains are characterized by significantly higher GM FD, lower WM FD, and a minimum in the GM-WM difference, in line with the findings in Figures 2 and 4. These developments were corroborated by SVR values (Extended Data Fig. 8b), which also showed numerical convergence in older infants and where the SVR of GM was lower and that of WM was higher in more mature brains. In light of the simulation study, these findings yield a

consistent geometric interpretation of the empirical age-FD effects: In terms of spatial scaling, GM develops from a more ‘plane-like’ geometry in younger infants (FD towards 2, SVR towards maximum) to a more ‘cube-like’ geometry in older infants (FD towards 3, SVR towards minimum). In contrast, WM geometry shows the opposite trend, developing from a more ‘cube-like’ behavior in younger infants to a more ‘plane-like’ behavior in older infants. In this context, it is useful to recall that this interpretation refers to the *dimension* of the structure rather than its *size*: While both GM and WM are naturally larger in older infants (cf. Extended Data Fig. 1), FD reflects the space-filling properties of these structures relative to the embedding 3D space, but regardless of absolute size.

Fifth, we ran a complementary control analysis to test if the inverse age-FD associations in WM could thus be artificially flipped by image manipulation (Extended Data Fig. 9). To this end, we hollowed out the WM volumes, effectively making them more similar to the cortical GM segmentations, as the GM-WM boundary closely follows the cortical ribbon (Extended Data Fig. 9a). In terms of the above geometric interpretation, we thus impose a more ‘plane-like’ geometry on the WM segmentations (because removing inner voxels shifts the SVR towards the plane), which does not equally affect the GM segmentations because the latter already show a more ‘plane-like’ behavior to begin with. Consequently, we hypothesized that the effect direction should uniquely flip for hollowed-out WM segmentations, but not for hollowed-out GM segmentations. Indeed, the direction of age effects remained positive for all GM areas, whereas it changed uniquely for those WM regions that showed inverse age associations when the original (full) segmentations were assessed. Notably, this held true for both regional parcels (Extended Data Fig. 9b) and global tissue segmentations (Extended Data Fig. 9c) in the dHCP, and this effect again replicated in the UCI validation data (Extended Data Fig. 9d).

Finally, we aimed to validate FD and volume against a biophysical proxy of WM microstructure (Extended Data Fig. 10). To this end, we computed the voxel-wise T1w/T2w ratio of the WM border[3,4] (Extended Data Fig. 10a-b) and observed that this measure was strongly and positively related to infant age (Extended Data Fig. 10c), consistent with previously reported dynamics of early-life WM development[5,6]. Notably, however, this microstructural proxy related significantly more strongly to WM FD than to WM volume (Extended Data Fig. 10d). Furthermore, partial correlations revealed

that the T1w/T2w-volume association vanished when controlling for FD, whereas the T1w/T2w-FD association persisted when controlling for volume, suggesting that FD captures unique aspects of WM development that are not accounted for by WM size alone.

Statistical details

Analyses on neonatal cortex morphology (Figure 3)

In the caption of Figure 3, *p*-values are provided in intervals for brevity, but exact values are listed here. Figure 3a shows the age-morphology associations of the neonatal cortex across eight measures: cortical thickness (Thick), curvature (Curv), gyrification index (GI), sulcation (Sulc), surface area (Surf), T1w/T2w ratio (T1/T2), volume (Vol), and fractal dimensionality (FD). For each region, the model with the highest R^2_{adj} was statistically compared to the second-best model with a permutation test (left panel): frontal left: FD (best) vs GI (second), $p_{perm} = 0$, $p_{FDR} = 0$; frontal right: FD vs GI, $p_{perm} = 0.009$, $p_{FDR} = 0.024$; anterior temporal lobe left: FD vs Surf, $p_{perm} = 0.005$, $p_{FDR} = 0.016$; anterior temporal lobe right: FD vs Surf, $p_{perm} = 0.028$, $p_{FDR} = 0.060$; anterior medial-inferior temporal gyrus left: GI vs FD, $p_{perm} = 0.418$, $p_{FDR} = 0.460$; anterior medial-inferior temporal gyrus right: GI vs FD, $p_{perm} = 0.232$, $p_{FDR} = 0.335$; posterior medial-inferior temporal gyrus left: FD vs GI, $p_{perm} = 0.050$, $p_{FDR} = 0.092$; posterior medial-inferior temporal gyrus right: FD vs GI, $p_{perm} = 4*10^{-4}$, $p_{FDR} = 0.002$; middle superior temporal gyrus left: FD vs Vol, $p_{perm} = 0.512$, $p_{FDR} = 0.512$; middle superior temporal gyrus right: FD vs Vol, $p_{perm} = 0.388$, $p_{FDR} = 0.458$; posterior superior temporal gyrus left: Vol vs FD, $p_{perm} = 0.056$, $p_{FDR} = 0.097$; posterior superior temporal gyrus right: FD vs Vol, $p_{perm} = 0.371$, $p_{FDR} = 0.458$; parahippocampal gyrus left: Surf vs Vol, $p_{perm} = 0.424$, $p_{FDR} = 0.460$; parahippocampal gyrus right: Vol vs Surf, $p_{perm} = 0.446$, $p_{FDR} = 0.464$; occipitotemporal gyrus left: Surf vs Vol, $p_{perm} = 0.015$, $p_{FDR} = 0.036$; occipitotemporal gyrus right: Surf vs FD, $p_{perm} = 0.257$, $p_{FDR} = 0.351$; occipital left: FD vs GI, $p_{perm} = 0$, $p_{FDR} = 0$; occipital right: FD vs GI, $p_{perm} = 0$, $p_{FDR} = 0$; insula left: Surf vs Vol, $p_{perm} = 0.007$, $p_{FDR} = 0.019$; insula right: Surf vs Vol, $p_{perm} = 0.303$, $p_{FDR} = 0.394$; anterior cingulate left: T1/T2 vs Vol, $p_{perm} = 1*10^{-4}$, $p_{FDR} = 4.3*10^{-4}$; anterior cingulate right: T1/T2 vs Vol, $p_{perm} = 0.203$, $p_{FDR} = 0.310$; posterior cingulate left: FD vs Vol, $p_{perm} = 0.067$, $p_{FDR} = 0.109$; posterior cingulate left: FD vs Vol, $p_{perm} = 0.050$, $p_{FDR} = 0.092$; parietal left: FD vs Vol, $p_{perm} = 0$, $p_{FDR} = 0$; parietal right: FD vs Vol, $p_{perm} = 0$, $p_{FDR} = 0$. For the analogous analyses in cortex-wide multiple linear models (Fig. 3b), all

comparisons of FD against the other measures resolved to zero for permuted RMSE (left) and R^2_{adj} (right).

Brain-to-brain comparisons across morphological measures (Figure 8)

Here we provide exact *p*-values for the genetic analyses in Figure 8c. For the twin-to-unrelated comparisons (left), the *t*-tests of the twin dissimilarity scores against zero resulted in the following values: FD, $p = 2.6*10^{-25}$, $p_{FDR} = 2.1*10^{-24}$; Curv, $p = 2.7*10^{-12}$, $p_{FDR} = 1.1*10^{-11}$; Vol, $p = 1.3*10^{-10}$, $p_{FDR} = 2.7*10^{-10}$; Thick, $p = 2.4*10^{-9}$, $p_{FDR} = 3.2*10^{-9}$; Surf, $p = 7.3*10^{-11}$, $p_{FDR} = 1.9*10^{-10}$; T1/T2, $p = 3.4*10^{-10}$, $p_{FDR} = 5.4*10^{-10}$; GI, $p = 0.018$, $p_{FDR} = 0.020$; Sulc, $p = 0.863$, $p_{FDR} = 0.863$. For the permutation tests of twin prediction accuracies (right), results were as follows: FD: overall / fraternal twins / identical twins: p_{perm} = 0 / 0 / 0; Curv: p_{perm} = 0 / 0.029 / 0; Vol: p_{perm} = 0 / 0.211 / 0; Thick: p_{perm} = 0 / 0.029 / 0; Surf: p_{perm} = 0 / 0.029 / 0; T1/T2: p_{perm} = 0 / 0.211 / 0; GI: p_{perm} = 0 / 0.029 / 0; Sulc: p_{perm} = 0.061 / 0.211 / 0.013.

References

1. Moog, N. K. *et al.* Intergenerational Effect of Maternal Exposure to Childhood Maltreatment on Newborn Brain Anatomy. *Biol. Psychiatry* **83**, 120–127 (2018).
2. Rasmussen, J. M. *et al.* Neuroanatomical Correlates Underlying the Association Between Maternal Interleukin 6 Concentration During Pregnancy and Offspring Fluid Reasoning Performance in Early Childhood. *Biol. Psychiatry Cogn. Neurosci. Neuroimaging* **7**, 24–33 (2022).
3. Makropoulos, A. *et al.* The developing human connectome project: A minimal processing pipeline for neonatal cortical surface reconstruction. *NeuroImage* **173**, 88–112 (2018).
4. Glasser, M. F. & Van Essen, D. C. Mapping human cortical areas in vivo based on myelin content as revealed by T1- and T2-weighted MRI. *J. Neurosci. Off. J. Soc. Neurosci.* **31**, 11597–11616 (2011).
5. Dubois, J. *et al.* The early development of brain white matter: A review of imaging studies in fetuses, newborns and infants. *Neuroscience* **276**, 48–71 (2014).
6. Grotheer, M. *et al.* Human white matter myelinates faster in utero than ex utero. *Proc. Natl. Acad. Sci.* **120**, e2303491120 (2023).
