# Supplementary material for: Fractal analysis of brain shape formation predicts age and genetic similarity in human newborns
Source: Nat Neurosci. 2025 Dec 29;29(1):171–85. doi: 10.1038/s41593-025-02107-w (PMC12779576; doi:10.1038/s41593-025-02107-w)
Supplement: Supplementary file 2 — Reporting Summary [file 41593_2025_2107_MOESM2_ESM.pdf]

nature portfolio

Corresponding author(s): Stephan Krohn
Carsten Finke

Last updated by author(s): Aug 14, 2025

Reporting Summary

Nature Portfolio wishes to improve the reproducibility of the work that we publish. This form provides structure for consistency and transparency in reporting. For further information on Nature Portfolio policies, see our Editorial Policies and the Editorial Policy Checklist.

Statistics

For all statistical analyses, confirm that the following items are present in the figure legend, table legend, main text, or Methods section.

n/a	Confirmed	
☐	☒	The exact sample size (*n*) for each experimental group/condition, given as a discrete number and unit of measurement
☐	☒	A statement on whether measurements were taken from distinct samples or whether the same sample was measured repeatedly
☐	☒	The statistical test(s) used AND whether they are one- or two-sided<br>*Only common tests should be described solely by name; describe more complex techniques in the Methods section.*
☐	☒	A description of all covariates tested
☐	☒	A description of any assumptions or corrections, such as tests of normality and adjustment for multiple comparisons
☐	☒	A full description of the statistical parameters including central tendency (e.g. means) or other basic estimates (e.g. regression coefficient) AND variation (e.g. standard deviation) or associated estimates of uncertainty (e.g. confidence intervals)
☐	☒	For null hypothesis testing, the test statistic (e.g. *F*, *t*, *r*) with confidence intervals, effect sizes, degrees of freedom and *P* value noted<br>*Give P values as exact values whenever suitable.*
☒	☐	For Bayesian analysis, information on the choice of priors and Markov chain Monte Carlo settings
☐	☒	For hierarchical and complex designs, identification of the appropriate level for tests and full reporting of outcomes
☐	☒	Estimates of effect sizes (e.g. Cohen's *d*, Pearson's *r*), indicating how they were calculated

Our web collection on statistics for biologists contains articles on many of the points above.

Software and code

Policy information about availability of computer code

Data collection	No software was used for the collection of data
Data analysis	Data analysis was implemented with R (versions 3.6.3 and 4.4.0) and MATLAB (versions 2017b, 2019b, and 2022b). For fractal analysis of structural brain segmentations, we used the openly available calcFD toolbox for MATLAB (https://github.com/cMadan/calcFD), modified to process neonatal neuroimaging data (see below for availability of custom code). Group-wise comparisons, correlation analyses, effect size calculations, cross-validation, random resampling, and multiple comparisons corrections were implemented with inbuilt facilities of R and MATLAB. For the statistical comparison of correlation coefficients, we used a MATLAB implementation of Williams' test (David M. Groppe, https://www.mathworks.com/matlabcentral/fileexchange/25984-r_test_paired) and the cocor package for R (version 1.1.4, http://comparingcorrelations.org/). The lmSupport package for R (version 2.9.13, https://rdrr.io/cran/lmSupport) was used to assess the hierarchical regression approach with F-tests for nested models. For the statistical assessment of clustering results, we used the sigclust package for R (version 1.1.0.1, https://cran.r-project.org/web/packages/sigclust/sigclust.pdf). Partial correlations were computed with the ppcor package for R (version 1.1). For the prediction of infant age, we used the PRISM toolbox for MATLAB (https://github.com/cMadan/prism) as well as the MATLAB functions 'fitlm' for simple multiple linear regression and 'fitrsvm' for support vector regression with a linear kernel. Analysis code supporting the findings of this study are available from the corresponding authors and the Open Science Framework (https://osf.io/6jck4/). Data visualization rests on ggplot functionalities in R as well as SurfIce (https://www.nitrc.org/projects/surfice/) with full-term equivalent meshes for brain mapping (https://brain-development.org, subcortical meshes were custom-made). Single points correspond to scans, infants, or brain regions, as indicated. Box-plots correspond to standard quantile-based display, showing median (central line), Q1-Q3 (box), with whiskers indicating 1.5*IQR from lower and upper hinges, with remaining data points considered outliers (https://ggplot2.tidyverse.org/reference/geom_boxplot.html).

For manuscripts utilizing custom algorithms or software that are central to the research but not yet described in published literature, software must be made available to editors and reviewers. We strongly encourage code deposition in a community repository (e.g. GitHub). See the Nature Portfolio guidelines for submitting code & software for further information.

Data

Policy information about availability of data

All manuscripts must include a data availability statement. This statement should provide the following information, where applicable:

- Accession codes, unique identifiers, or web links for publicly available datasets
- A description of any restrictions on data availability
- For clinical datasets or third party data, please ensure that the statement adheres to our policy

All data analyzed in the main text were obtained from the neonatal release of the developing Human Connectome Project (dHCP; Edwards et al., 2022; www.developingconnectome.org), publicly available through the NIMH data portal (https://nda.nih.gov/edit_collection.html?id=3955). Replication and validation analyses (Extended Data Fig. 5, 7, and 9) were implemented in a second external dataset from the University of California, Irvine (UCI; Rasmussen et al., 2022), publicly accessible through the NIMH Data Archive Collection #1890 (https://nda.nih.gov/edit_collection.html?id=1890).

Research involving human participants, their data, or biological material

Policy information about studies with human participants or human data. See also policy information about sex, gender (identity/presentation), and sexual orientation and race, ethnicity and racism.

Reporting on sex and gender	The biological sex of the infants was recorded by the dHCP and explicitly analyzed in Figure 4 and Extended Data Figures 4 and 14.
Reporting on race, ethnicity, or other socially relevant groupings	An analysis of socially relevant groupings was outside the scope of the current study.
Population characteristics	Population characteristics of the dHCP data (discovery) and the UCI data (validation) are reported in the Methods. In brief, there were n=782 infants in the dHCP data (360 females, 422 males). Mean birth age in the dHCP was 37.89 ± 4.17 postmenstrual weeks [range: 23.0 – 43.57], and age at first scan was 39.81 ± 3.55 weeks [range: 26.71 – 45.14]. Of these dHCP infants, 682 were born from singleton pregnancies, while 100 were born from multifetal pregnancies. Genetic analyses were based on single nucleotide polymorphisms array genotype data. In the UCI data, there were n=99 infants (48 females, 51 males) with age at scan 42.87 ± 2.01 weeks [range: 39.57 – 48.57].
Recruitment	Recruitment of dHCP infants was conducted at St Thomas' Hospital, London (Edwards et al., 2022); families received reimbursement of travel expenses. Recruitment of UCI infants was conducted at the University of California, Irvine (Rasmussen et al., 2022); families received 100 USD for participating in the MRI session.
Ethics oversight	dHCP: United Kingdom Health Research Authority (Research Ethics Committee reference number: 14/LO/1169); UCI: Institutional Review Board IRB #2009-7251.

Note that full information on the approval of the study protocol must also be provided in the manuscript.

Field-specific reporting

Please select the one below that is the best fit for your research. If you are not sure, read the appropriate sections before making your selection.

☒ Life sciences ☐ Behavioural & social sciences ☐ Ecological, evolutionary & environmental sciences

For a reference copy of the document with all sections, see nature.com/documents/nr-reporting-summary-flat.pdf

Life sciences study design

All studies must disclose on these points even when the disclosure is negative.

Sample size	A-priori sample size calculation was not implemented; however, we here analyze the dHCP data which constitutes one of the largest neonatal cohorts ever collected (n=782 infants), greatly exceeding typical sample sizes in perinatal neuroimaging. Furthermore, we analyze a second dataset for validation (UCI) with n=99 additional infants.
Data exclusions	No primary data were excluded. For the twin analyses, seven twin pairs had to be discarded for a subset of the analyses, one because no age matches of unrelated infants were available, and six because the two twin siblings themselves were scanned more than one day apart, as detailed in the Methods.
Replication	All findings here are based on computational analysis, not experimental intervention, such that experimental replication is not applicable. However, we implement several replication and validation analyses in an indepdent external dataset (UCI), as shown in Extended Data Figures 5, 7, and 9. Moreover, we applied cross-validation, random resampling, and different model types to ensure replication across variations in input data and analytical approaches. Reproducibility is also supported by the OSF repository.
Randomization	Randomization is not applicable, as participants were not assigned to experimental groups. Group comparisons rest on demographic, developmental, and genetic factors intrinsic to the participants.

Blinding	Blinding to group assignment was not possible, but also not applicable to the current study. For spatial analysis, however, the lead researcher (SK) was initially blinded to the ROI indices (i.e., which number corresponds to which brain region).

Reporting for specific materials, systems and methods

We require information from authors about some types of materials, experimental systems and methods used in many studies. Here, indicate whether each material, system or method listed is relevant to your study. If you are not sure if a list item applies to your research, read the appropriate section before selecting a response.

Materials & experimental systems

n/a	Involved in the study
☒	☐ Antibodies
☒	☐ Eukaryotic cell lines
☒	☐ Palaeontology and archaeology
☒	☐ Animals and other organisms
☒	☐ Clinical data
☒	☐ Dual use research of concern
☒	☐ Plants

Methods

n/a	Involved in the study
☒	☐ ChIP-seq
☒	☐ Flow cytometry
☐	☒ MRI-based neuroimaging

Plants

Seed stocks	*Report on the source of all seed stocks or other plant material used. If applicable, state the seed stock centre and catalogue number. If plant specimens were collected from the field, describe the collection location, date and sampling procedures.*
Novel plant genotypes	*Describe the methods by which all novel plant genotypes were produced. This includes those generated by transgenic approaches, gene editing, chemical/radiation-based mutagenesis and hybridization. For transgenic lines, describe the transformation method, the number of independent lines analyzed and the generation upon which experiments were performed. For gene-edited lines, describe the editor used, the endogenous sequence targeted for editing, the targeting guide RNA sequence (if applicable) and how the editor was applied.*
Authentication	*Describe any authentication procedures for each seed stock used or novel genotype generated. Describe any experiments used to assess the effect of a mutation and, where applicable, how potential secondary effects (e.g. second site T-DNA insertions, mosiacism, off-target gene editing) were examined.*

Magnetic resonance imaging

Experimental design

Design type	Structural MRI
Design specifications	Anatomical acqusition, no task design
Behavioral performance measures	Not studied here

Acquisition

Imaging type(s)	Structural T2-weighted images
Field strength	3T
Sequence & imaging parameters	dHCP after Edwards et al. (2022): T2-weighted images were acquired using a Fast Spin Echo sequence in sagittal and axial slice stacks with in-plane resolution 0.8×0.8mm^2 and 1.6mm slices, overlapped by 0.8mm; TR/TE = 12000/156ms; UCI after Rasmussen et al. (2022): T2-weighted images acquired using a Turbo Spin Echo sequence with TR/TE=3200/255ms, matrix=256x256x160, resolution=1x1x1mm^3.
Area of acquisition	Whole-brain acquisition
Diffusion MRI	☐ Used ☒ Not used

Preprocessing

Preprocessing software	Preprocessed data were included as provided by the dHCP standardized preprocessing pipelines, following the minimal processing pipeline for neonatal cortical surface reconstruction (Makropoulos et al., 2018). Brain segmentations in the dHCP rest on the DrawEM algorithm (https://github.com/MIRTK/DrawEM). UCI preprocessing was implemented with custom modifications of the Human Connectome Project anatomical pipeline (Rasmussen et al., 2022).

Normalization	Images were transformed using age-specific normative templates (see below).
Normalization template	Age-specific week-wise templates as provided with the dHCP (https://brain-development.org); age-specific NIH pediatric templates for UCI.
Noise and artifact removal	dHCP: Motion correction after Cordero-Grande et al., 2018; Makropoulos et al., 2018; UCI: prospective motion correction with volumetric navigators; ANT DenoiseImage and N4BiasFieldCorrection (Rasmussen et al., 2022).
Volume censoring	None

Statistical modeling & inference

Model type and settings	ROI-wise analyses are univariate group comparisons or continuous models; predictive models for age are different types of regression models (relevance vector, simple multiple linear, support vector).
Effect(s) tested	No task or stimulus conditions applicable. Effects tested include group differences, age associations, prediction accuracy (mean absolute error for continuous prediction, accuracy for categorical predictions). Effect sizes of correlational analyses were statistially compared with tests for dependent or independent groups, as applicable, using the cocor package for R.

Specify type of analysis: ☐ Whole brain ☒ ROI-based ☐ Both

Anatomical location(s)	Anatomical locations are based on the modified ALBERT atlas for neonatal brain anatomy (Gousias et al., 2012; Makropoulos et al., 2014).
Statistic type for inference (See Eklund et al. 2016)	ROI-wise inference
Correction	FDR / permutation

Models & analysis

n/a	Involved in the study
☒	☐ Functional and/or effective connectivity
☒	☐ Graph analysis
☐	☒ Multivariate modeling or predictive analysis

Multivariate modeling and predictive analysis	Independent variables for predictive analyses included ROI-wise fractal dimensionality values (observations x regions), volumes, or both. Dimensionality reduction in the main analyses was implemented with Principal Component Analysis. Cross-validation was implemented with a 10-fold cross-validation scheme with random repetitions. Evaluation metrics were mean absolute prediction error and variance explained in unseen data. Evaluation of twin predictions was based on a custom rank loss measure, prediction accuracy, and null distribution testing through permutation, as detailed in the Methods. The identical approach was applied for the comparative analyses using surface-derived morphological measures (e.g., Fig. 8).
